# Driven Self-Assembly of Patchy Particles Overcoming Equilibrium Limitations

**DOI:** 10.1021/acs.jctc.4c01118

**Published:** 2024-09-10

**Authors:** Shubhadeep Nag, Gili Bisker

**Affiliations:** † Department of Biomedical Engineering, Faculty of Engineering, Tel Aviv University, Tel Aviv 6997801, Israel; ‡ The Center for Physics and Chemistry of Living Systems, Tel Aviv University, Tel Aviv 6997801, Israel; ¶ The Center for Nanoscience and Nanotechnology, Tel Aviv University, Tel Aviv 6997801, Israel; § The Center for Light-Matter Interaction, Tel Aviv University, Tel Aviv 6997801, Israel; ∥ The Center for Computational Molecular and Materials Science, Tel Aviv University, Tel Aviv 6997801, Israel

## Abstract

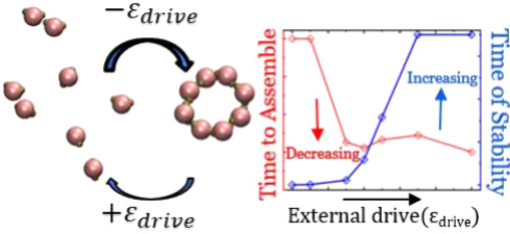

Bridging biological complexity and synthetic material design, we investigate dissipative self-assembly in patchy particle systems. Utilizing Monte Carlo and Molecular Dynamics simulations, we demonstrate how external driving forces mitigate equilibrium trade-offs between assembly time and structural stability, traditionally encountered in self-assembly processes. Our findings also extend to biological-mimicking environments, where we explore the dynamics of patchy particles under crowded conditions. This comprehensive analysis offers insights into advanced material design, opening avenues for innovations in nanotechnology applications.

The exploration of complex biological processes and the development of self-assembling materials, ranging from protein and oligonucleotide assembly to liquid crystal and gel formation, has significantly advanced our understanding of the thermodynamics of structure-forming systems, aiding biomimetic material synthesis.^1−10^ In the realm of thermodynamics, processes that persistently utilize external energy sources are termed dissipative processes. As not all of the energy is converted into work, the dissipated energy results in the production of entropy, and the system is essentially operating under nonequilibrium conditions.^11^ In order to maintain the functionality of biological systems, numerous life-sustaining functions operate far-from-equilibrium, driven by continuous energy consumption.^12,13^ A noteworthy example is the formation of actin filaments and microtubules from the assembly of their respective monomers, actin, and tubulin,^9,14,15^ through the hydrolysis of triphosphate to diphosphate nucleotide. These assemblies are categorized as “dissipative self-assembly” (DSA).

A deep comprehension of the principles and mechanisms underlying DSA is pivotal for understanding the essential processes of life. Furthermore, this knowledge forms the foundation for the development of synthetic systems that mimic the dynamic and adaptable properties of biological assemblies, holding tremendous potential for the creation of responsive nanodevices.^16−19^ The principles of DSA, extended to DNA and RNA engineering, offer precise control in nanotechnology applications, including therapeutics and biosensing.^20−24^

DSA processes, operating far from equilibrium, can circumvent the limitations of equilibrium self-assembly, such as balancing kinetic control with thermodynamic stability, crucial for designing efficient nanostructures and understanding biological phenomena.^25^ In colloidal polymers, the increase in particle diversity complicates rapid folding and achieving the desired target, especially with orientation-dependent interactions that are critical in larger systems.^26^ Patchy particles, embodying systems with such interactions, have attracted considerable interest due to their versatile applications and innovative synthesis methods like colloidal fusion and evaporative deposition.^27−30^ In computational studies, one of the widely employed models for simulating patchy particles is Kern and Frenkel’s model,^31^ which have been developed further by subsequent studies,^32−34^ has been pivotal in understanding critical phenomena such as the stability of liquid phase in patchy colloids, liquid–liquid phase separation in membraneless organelles, liquid–vapor coexistence region in patchy liquids, etc.^35−37^

A recently developed model focused on rapidly achieving stable target structures from randomly oriented particles in a model of lattice gases, by integrating an external drive into the self-assembly process.^38^ This drive manipulated the interaction energy between particles that were nearest neighbors in the target structure. If such particles approached each other, the drive lowered their interaction energy, favoring the acceptance of their movement toward the assembly. Conversely, if the nearest neighbors attempted to move apart, the drive penalized their interaction energy, discouraging the disassembly move. This approach mimics the directed self-assembly seen in experimental systems such as DNA nanostructures,^39−41^ and colloidal crystals,^42^ where selective interactions and external stimuli such as light, pH, and electromagnetic field promote the desired configurations. Such application of an external drive nudges the system toward self-assembly and helps sustain the assembled structure, necessitating a constant energy source throughout the simulation, akin to biological mechanisms where ATP and GTP facilitate the polymerization of actin filaments and microtubules. This approach, demonstrated through 2D lattice models and MC simulations, illustrates how nonequilibrium conditions can facilitate the formation of more stable structures by bypassing the usual speed-accuracy trade-offs seen in equilibrium systems.^38,43^ To refine the model, the stochastic landscape method, which was introduced in a follow-up study, enhanced the predictions of initial assembly times and improved control over nonequilibrium self-assembly.^44^ It is worth noting that other models also contribute to a broader understanding of dissipative self-assembly mechanisms.^45−48^

Despite validating nonequilibrium self-assembly models in 2D lattice MC simulations,^38,44^ their extension to 3D systems remains unexplored. To address this, we have selected the patchy particles model to examine self-assembly, with a target structure of a ring. Simulating ring-like assemblies is inspired by various engineered self-assembly processes, such as the formation of DNA/RNA rings and cyclic peptides that evolve into supramolecular tubules, with significant applications in fields ranging from electronics to therapeutic drug delivery.^49−53^ Moreover, the self-assembly of tubulin into ring-shaped microtubules further underscores the relevance of our focus on ring-shaped systems.^54,55^

In this study, we tackle the inherent trade-offs observed in equilibrium simulations of self-assembling systems comprising 8 patchy particles in 3D. Utilizing Equilibrium Monte Carlo (MC) simulations, we first analyze the self-assembly of the particles in both identical and two-state configurations, forming an 8-sided polygon. Our focus is on understanding the stability and formation time of these structures, with respect to the interaction energy between patches. To overcome the equilibrium trade-offs, we introduce a nonequilibrium external bias, favoring desired nearest neighbors interactions. Our thermodynamic analysis includes entropy and an order parameter introduced to track the transition from disordered to organized states. To demonstrate the robustness of our design principles, we simulated a large system of 1000 particles with similar patch orientations to those of the patchy particles, with one or two states, forming an octagon. We also study the self-assembly of an 8-patchy particles system with 48 crowding agents to mimic more realistic scenarios, where the crowding agents represent the solvents or surrounding cellular components. We employ both MC and Molecular Dynamics (MD) simulations with and without the nonequilibrium drive, confirming the effectiveness of external bias in mitigating equilibrium trade-offs. Our MD simulation with real-time dynamics complements our MC results and lays the path for future studies with diverse systems.

## Computational Details

We focus on systems comprising 8 patchy particles, each adorned with two strategically placed patches with an angle of 135-degree relative to the particle core (Figure S1 in the Supporting Information (SI)). One type of system consists of identical particles with a single state, α, while the other type consists of particles with two different states, α and β. This distinction is inspired by biological parallels, namely, actin filaments in which the G-actin monomers are of similar types,^56^ and microtubules in which the tubulin building blocks consist of two different types, α- and β-tubulin.^57^ The simulation of the patchy particle system is conducted using the Kern-Frenkel model^31^ (Subsection S1.1 in the SI).

**Figure 1:**
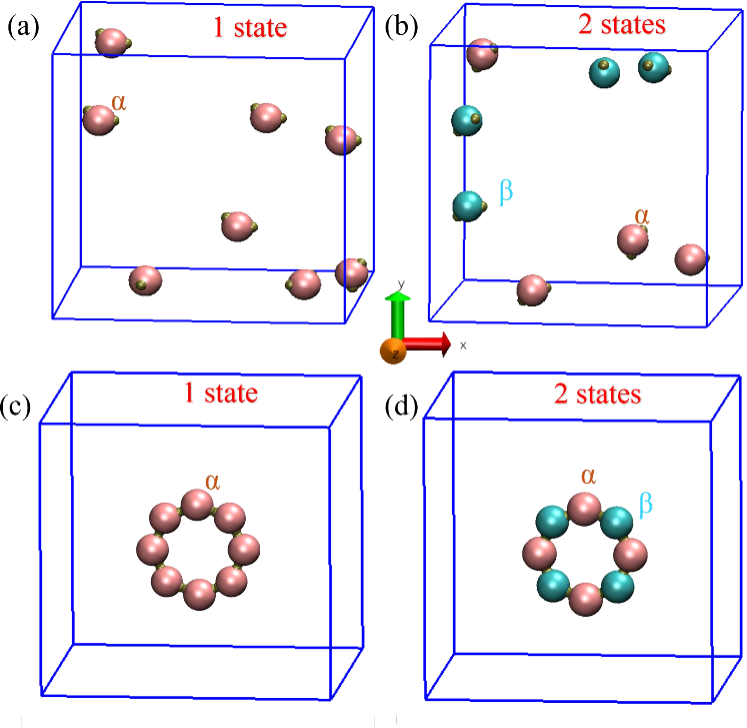
(a) Random initial configurations for 8 patchy particle of the one-state (α) system, and (b) the two-state (α and β, pink and cyan, respectively) system. (c) The configuration of the target structure of the one-state system, and (d) the two-state system.

A random initial configuration of both these systems is shown in Figures 1(a) and 1(b), respectively. The patches are designed to facilitate the formation of a ring-like structure or a polygon, encompassing all 8 particles. For the system of the single state α, all particles are potential nearest neighbors (Figure 1(c)), thus having identical interaction energies. Conversely, in the two-state system, the target ring is defined such that nearest neighbor pairs form only between different state particles, α and β (Figure 1(d)) while same-state pairs (α–α or β–β) have no energetic interaction.

To examine how the introduction of external bias mitigates these limitations, our study concentrates on two metrics, namely, the time required to form the first assembled structure, denoted as *T*_*fas*_, and the time the system remains in the assembled state as a proxy for the structural stability of the target, represented as *T*_*stable*_. Quantification of *T*_*fas*_ and *T*_*stable*_ is based on simulations initiating with the 8 patchy particles in random orientations, or at the target structure, respectively. The details of the MC and MD simulations, both without and with an external driving force, and the physical origin of the external drive are provided in Subsections S1.2, S1.3, and S1.4 of the SI, with Figure S2 illustrating the effect of the external driving force on the assembly protocol.

## Results and Discussion

### Equilibrium MC Simulations

**Figure 2:**
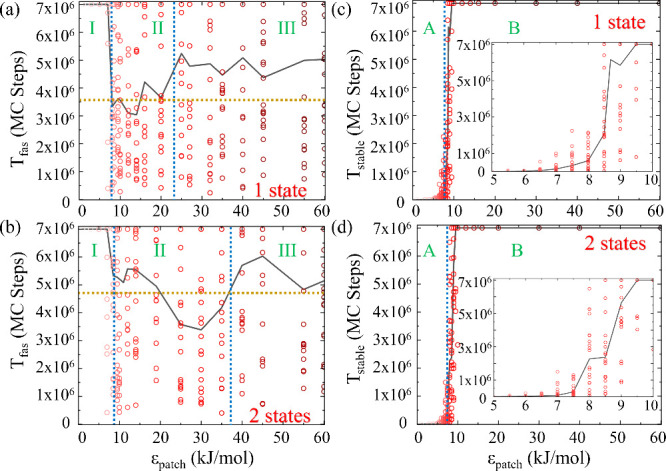
Dynamics of 8 patchy particle systems. (a) Median *T*_*fas*_ values (black line), alongside individual simulation results (red circles), for various ϵ_*patch*_ values for the one-state system, and (b) two-state system. The ϵ_*patch*_ range is divided into 3 regions marked by blue dotted lines, where the average value of *T*_*fas*_ over the median values in the intermediate region II is shown in a golden dotted line. (c) Variation of median values of *T*_*stable*_ (black line), alongside individual simulation results (red circles), as a function of patchy interactions for the one-state system, and for the (d) two-state system. The ϵ_*patch*_ range is divided into 2 distinct regions marked by a blue dotted line. Together, these figures underscore the influence of the interaction energy between patches on the assembly efficiency and stability of the system.

We begin with a comprehensive analysis of systems comprising 8 patchy particles with patchy interaction energies, ϵ_*patch*_, ranging from 0 to 60 kJ/mol using MC simulations at 65 K. In Figures 2(a) and 2(b), we illustrate the variation in median *T*_*fas*_ values (black line) among 20 distinct simulations (red circles) across a range of ϵ_*patch*_ values, for systems of 8 patchy particles with one and two internal states, respectively, under equilibrium conditions.

For the one-state system (Figure 2(a)), we observe a constant *T*_*fas*_, equivalent to the total number of MC steps, for low ϵ_*patch*_ values ranging from 0 to 7 kJ/mol. Similarly, for the two-state system (Figure 2(b)), this range extends from 0 to 8.5 kJ/mol. At these low interaction energies, the particles struggle to coalesce into a stable assembly, indicated by the inability of most simulations to reach the target structure within the simulation time. As ϵ_*patch*_ increases, particularly in the intermediate range of 8 to 25 kJ/mol for the one-state system and 9 to 35 kJ/mol for the two-state system, *T*_*fas*_ decreases, reaching a lower value and then fluctuating within this range. Here, the average *T*_*fas*_ values for the one and two-state systems are approximately 3.5 × 10^6^ and 4.7 × 10^6^ MC steps, respectively, as highlighted by the golden dotted lines in both figures. This region is thus identified as the optimal energy regime for assembly, where most simulations successfully achieve the target structure. The slightly higher average value of *T*_*fas*_ in this range for the two-state system shows the inherent difficulties posed by the selective interactions present in this case. With a further increase in ϵ_*patch*_, *T*_*fas*_ begins to rise again, suggesting that overly strong interactions may hinder efficient assembly due to kinetic traps. In this high-energy range, *T*_*fas*_ reaches a plateau around 5 × 10^6^ MC steps for the one-state system and 5.7 × 10^6^ for the two-state system, indicating a diminishing efficiency in the assembly process due to high interaction energies.

In our analysis, the behavior of *T*_*fas*_ across varying ϵ_*patch*_ values allows us to categorize the interaction energy range into three distinct regions (Figures 2(a) and 2(b), blue dotted lines). Region I encompasses the low ϵ_*patch*_ range. Here, the relatively high *T*_*fas*_ values are attributed to insufficient interaction energy to counteract thermal perturbations, and the assembly is delayed (Movies S1 and S2 in the SI). Region II represents the intermediate ϵ_*patch*_ range, where a noticeable decrease in *T*_*fas*_ is observed, signifying enhanced assembly efficiency. Region III, comprising the high ϵ_*patch*_ range, is characterized by an increase in *T*_*fas*_, on average, indicating that the strong interactions result in kinetic traps at local minima and an overall slower assembly.

The variation of *T*_*stable*_, representing the durability of the target structure across different ϵ_*patch*_ values, reveals the impact of patchy interaction on structural stability. For each set of simulations, we determined the median *T*_*stable*_ values starting from the target configuration (Figures 2(c) and 2(d), black line). Upon examining the plots for both one and two-state systems, we can categorize the results into two distinct regions, labeled as A and B. In the lower ϵ_*patch*_ range, both systems exhibit lower *T*_*stable*_ values, underscoring the ineffectiveness of weak interaction energies in sustaining the target structure over time. Beyond a critical ϵ_*patch*_ threshold, of approximately 8 kJ/mol for both systems, *T*_*stable*_ values begin to increase, suggesting that higher interaction energies are effective in stabilizing the target structure.

In the equilibrium self-assembly of our system, the contrasting dynamics across different regions of interaction energy (ϵ_*patch*_) emphasize the effect an external bias could have. Our focus is on Regions I and A, which are characterized by longer *T*_*fas*_ and low *T*_*stable*_. In this case, the employed external bias can be most effectively applied since it can potentially enhance both the rate of assembly and the stability of the formed structures. By targeting Regions I and A, with a driving mechanism that favors nearest-neighbor interactions, our approach leverages external bias to overcome challenges in the self-assembly process.

### Nonequilibrium MC Simulations

**Figure 3:**
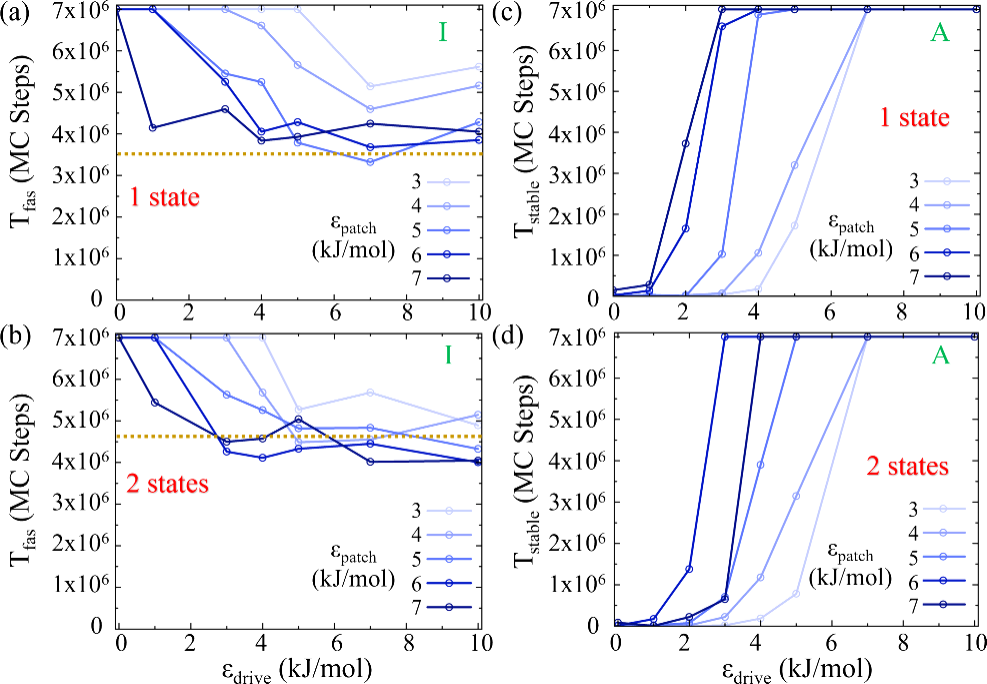
Effect of external bias between the patches on the variation of *T*_*fas*_ for (a) one-state system and (b) two-state system, in their respective regions I. (c) Variation of *T*_*stable*_ in the presence of bias for one-state system and (d) two-state system, in their respective regions A. For increased patchy interactions, smaller values of external bias between patches result in a faster-reaching assembly structure and increased stability of the target structure. Each value is the median of 20 distinct simulations.

The effects of the external bias on both *T*_*fas*_ and *T*_*stable*_ for a system of 8 patchy particles with one and two internal states for a range of patchy interaction energies within region I and A is presented in Figure 3. For both the one-state system (Figure 3(a)) and the two-state system (Figure 3(b)), we observe that an external drive up to 10 kJ/mol consistently aids in self-assembly by reducing *T*_*fas*_ for ϵ_*patch*_ values ranging from 3 to 7 kJ/mol. Interestingly, at the lowest value tested of ϵ_*patch*_ of 3 kJ/mol, *T*_*fas*_ does not improve until the external drive exceeds 7 kJ/mol for the one-state system and 5 kJ/mol for the two-state system. This indicates a threshold effect where the external drive becomes significant enough to influence assembly kinetics.

When ϵ_*patch*_ is increased to a large value of 7 kJ/mol, even a modest external drive is found to be sufficient to steer the system toward the target structure efficiently (Movies S3 and S4 in the SI). However, beyond the initial reduction observed for any value of patchy interaction ϵ_*patch*_, further increases in the external drive do not continue to lower *T*_*fas*_, suggesting a saturation point where the system’s response to ϵ_*drive*_ plateaus. This behavior is visualized by the golden dotted lines in Figures 3(a) and 3(b), which are drawn at 4.7 × 10^6^ and 3.5 × 10^6^ MC steps, respectively—consistent with the equilibrium *T*_*fas*_ averages observed in Region II of the equilibrium simulation.

The influence of external drive on the stability of the assembled structure is depicted in Figures 3(c) and 3(d), for systems of 8 patchy particles with one and two-state configurations, respectively. An increase in ϵ_*drive*_ correlates with an increase in *T*_*stable*_ for both cases. This indicates that the external drive not only expedites the assembly process but also reinforces the stability of the resulting structure. For systems with lower patch interaction energy (3 kJ/mol), a substantial external drive is necessary to surpass the stability threshold. However, as the patch interaction energy increases, a smaller external drive suffices to achieve structural stability for both one and two-state systems.

This optimization in nonequilibrium simulations provides a promising method to surpass the limitations encountered in equilibrium scenarios, where high *T*_*fas*_ and low *T*_*stable*_ in regions I and A can hinder practical applications. By carefully calibrating the external drive, it is possible to direct the system toward rapid and stable assembly, effectively leveraging the nonequilibrium dynamics to mitigate the challenges of self-assembly in complex systems. The application of such external drive in this region, capable of enhancing the system’s stability and expediting its assembly, provides a significant insight for designing self-assembling systems where the interplay between kinetic efficiency and structural durability is essential. To ensure the biological relevance of our findings, we conducted additional simulations at 300 K, confirming that our key results, including increased stability and assembly rate in the presence of an external drive, remain consistent at higher temperatures (see Figure S3 and Section S2 in the SI). Furthermore, by carrying out simulations of large systems with periodic boundaries, we demonstrated the merit of our approach across different conditions (see Figure S4 and Section S3 of the SI.).

In our investigation of the nonequilibrium assembly kinetics, we track the assembly process by an order parameter, *R*, defined as the ratio of the system’s instantaneous number of virtual bonds between the patches to the corresponding total number of bonds between patches in the target state (Figure 1(c) and 1(d)), as well as the entropy production, *S*. We computed these quantities for the system with patchy interaction energy of 4 kJ/mol of regions I and A and external drive of 4 kJ/mol.

**Figure 4:**
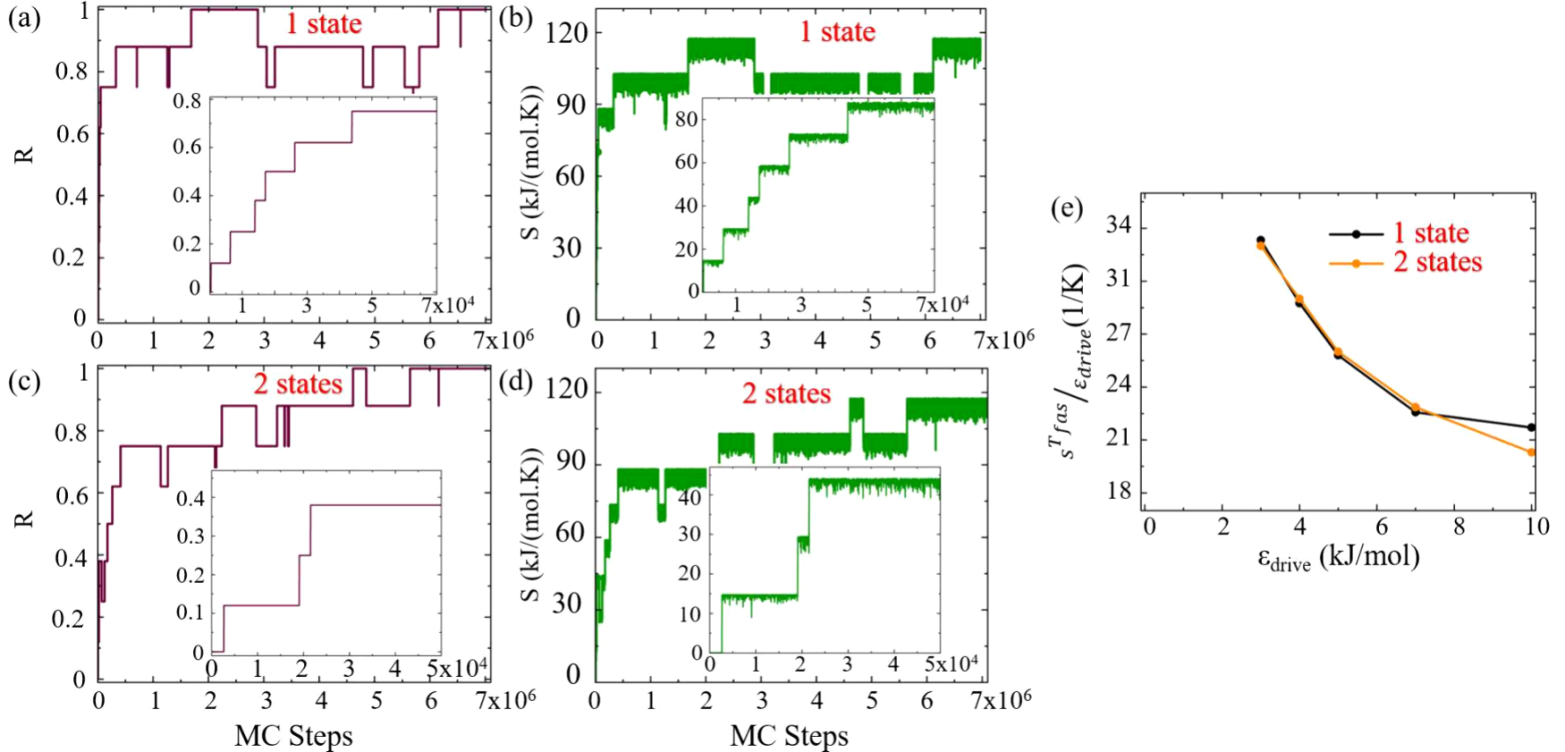
(a) The order parameter, *R*, as a function of MC steps of the one-state system. (b) The entropy production, *S*, as a function of MC steps of the one-state system. (c) The order parameter, *R*, as a function of MC steps of the two-state system. (d) The entropy production, *S*, as a function of MC steps of the two-state system. The results are for a single MC realization under nonequilibrium drive (ϵ_*patch*_ = 4 kJ/mol and ϵ_*drive*_ = 4 kJ/mol). The inset plots are zoom-in into the early simulation steps. (e) The total entropy production up to the first assembly time normalized by the external driving energy, ϵ_*drive*_, as a function of ϵ_*drive*_ for the one- (black) and two-state (orange) systems.

The value of *R* for a single-state system under the influence of an external drive as a function of the MC steps is illustrated in Figure 4(a) for a representative realization. A perfect assembly corresponds to 8 times the patch interaction energy, with any deviation leading to a proportional decrease in total energy. Since the number of bonds is discrete, the order parameter is quantized, incrementing in steps of 1/8, from 0 (no bonds) to 1 (all 8 bonds formed), reflecting the system’s evolution toward full assembly. Under nonequilibrium conditions, there is a rapid structural assembly within a few thousand MC steps - an observation absent in the equilibrium case (Figure S5(a) of SI). For the same realization, the overall increase of *S* as a function of MC steps, depicted in Figure 4(b), further underscores the nonequilibrium nature of the process, contrasting the vanishing entropy production rate seen in Figure S5(b) of the SI for a similar system in equilibrium. For a particular realization, the behavior of *R* as a function of the MC steps for the two-state system, displayed in Figure 4(c), mirrors the single-state scenario, similarly to the entropy production during the assembly process depicted in Figure 4(d). These are also contrasted with the respective equilibrium data provided in Figures S5(c) and S5(d) in the SI, where in the absence of a drive, there are fluctuations in *R* mostly around 0, 1/8, and 2/8, and no entropy production.

Comparing the one- and two-state systems, it is evident that the former stabilizes more rapidly, as manifested in the behavior of the value of *R* (Figure S6(a) in the SI), and the value of *S* (Figure S6(b) in the SI). In the two-state case, both parameters exhibit initial fluctuations before showing a rapid increase, indicative of more complex assembly dynamics. This is further evidenced by the total energy fluctuations for different interaction values in Figure S7 of the SI, showcasing a more rugged energy landscape and slower assembly process for the two-state system toward target structure formation, compared to the smoother trajectory and expedited assembly progression of the one-state system (see Section S4 in the SI). Our simulation results align with previous findings using 2D lattice-model systems.^38,43,44^

The total entropy produced up to the first assembly time normalized by the value of the drive ϵ_*drive*_ as a function of the drive value is presented in Figure 4(e) for a representative realization. This normalized entropy production could be used as a proxy to the number of entropy-producing cycles, as in each MC step in which the drive has an effect, the contribution of the external drive to the total entropy production is of the order of the drive value itself. Evidently, as ϵ_*drive*_ increases, the number of entropy production cycles decreases, indicating a more efficient assembly. The key findings from the simulation of 1000 patchy particles are consistent with our findings of 8 patchy particles (see Figures S8–S13, Movies S5 and S6, and Section S5 in the SI).

### Nonequilibrium MC and MD Simulations in a Crowded Environment

Our simulations of an 8-patchy particle system have revealed significant insights into self-assembly mechanisms, crucial in both biological systems and nanotechnology, particularly in gaseous-like environments where particles face vast spaces and infrequent interactions. Nevertheless, in many scenarios, particles must navigate a crowded environment. We, therefore, have progressed to simulating a more complex system, of the one-state case, where we have included 48 crowding particles in addition to the 8 patchy particles. This reflects a more realistic biological context, akin to intracellular conditions with densely packed cytoplasm and frequent interactions.^58,59^ The “crowding effect” plays a critical role, altering particle movement and interaction dynamics, thus emulating conditions like the cellular interior,^60^ allowing us to explore the effects of crowding on the stability and morphology.^61^

**Figure 5:**
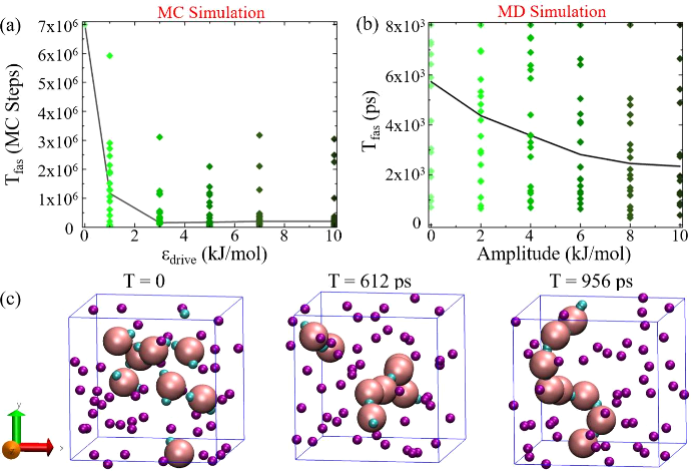
Simulations in a crowded environment at ϵ_*patch*_ of 3.7 kJ/mol. (a) Variation of *T*_*fas*_ under nonequilibrium conditions as a function of the external drive value in MC simulations, and (b) as a function of the amplitude of the external time-dependent square potential with a periodicity of 20 ps in MD simulations. Each dot represents an individual realization, while the black curve is the median value. (c) Snapshots from the MD simulation of 8 patchy particles in the presence of 48 crowd agents (shown in purple), with patch interaction energy of ϵ_*patch*_ = 3.7 kJ/mol and external square wave drive amplitude of 8 kJ/mol, captured at the beginning of the simulation, midway through the formation process (612 ps), and upon achieving a fully formed structure (956 ps). While the crowding agents and patchy particle beads have the same van der Waals radii, the crowding agents’ size is reduced by one-third in the snapshot to enhance target formation visualization.

In a comparative analysis of MC and MD simulations for this system, we focused on the effect of external drive when the equilibrium limitations are prominent, and chose an interaction energy of ϵ_*patch*_ = 3.7 kJ/mol from regions I and A of *T*_*fas*_ and *T*_*stable*_ plots (Figure 2). In the MC simulations, we observe a significant decrease in the time to fastest assembly, *T*_*fas*_, from 7 × 10^6^ to ∼2 × 10^5^, in the presence of the driving forces (Figure 5(a)), indicating an acceleration in the assembly process when the external bias is increased from 0 kJ/mol (representing no external bias) to 3 kJ/mol. However, for ϵ_*drive*_ > 3 kJ/mol, there is no substantial improvement in *T*_*fas*_ with further increase in bias up to 10 kJ/mol, consistent with our previous findings in the one-state 8-particle system without crowd agents. The median value of *T*_*fas*_, plotted as a black line, confirms this trend, demonstrating a sharp reduction in *T*_*fas*_ at lower external drive strengths, which plateaus at higher values.

In MD simulations, there is also a tendency for reduced assembly time with the introduction of the external drive. The external drive is applied in the form of a time-dependent square wave potential^62^ inspired by biological events and experimental studies.^63−65^ The applied external drive, analogous to an electromagnetic field, alters the patchy potential by introducing an additional time-varying component. The square wave amplitude is varied at a periodicity of 20 ps, resulting in a higher and lower potential with respect to a baseline of 3.7 kJ/mol (Figure S14 in the SI). Here, the increase in the amplitude of the periodic square wave resembles the increase in the absolute value of the external drive used in MC simulations. Indeed, when the amplitude is increased from 0 (equilibrium) to 4 kJ/mol, the median of *T*_*fas*_ decreases from 5731 to 3751 ps (Figure 5(b)). As we continue to increase the amplitude up to 10 kJ/mol, *T*_*fas*_ eventually stabilizes around ∼2400 ps. This variation confirms that our results are consistent with those from our MC simulations, validating our approach of using the time dependent square wave potential. In a concurrent study,^62^ we show that the periodic square wave effectively enhances the forces between particles. Further, when varying the periodicity of the square wave for 8 patchy particles with 48 crowds from 20 to 40 ps, the *T*_*fas*_ only reduces to ∼4000 ps (Figure S15 in the SI), probably due to the less frequent force enhancements between the patches (Section S6 in the SI). Similar studies were carried out for the same crowd system with patchy particles of two states, showing consistent results (see Figure S16 in the SI). The detailed mechanism of facilitating bond formation between patchy particles in the presence of a time-dependent square wave is articulated in Figure S17 of the SI.

Figure 5(c) presents snapshots from an MD simulation of the patchy particle system with crowds under nonequilibrium conditions, capturing the evolution toward self-assembly. While the initial snapshot at *T* = 0 ps shows a disordered state with dispersed particles and crowders, by 612 ps, owing to the square wave potential, the particles start to aggregate toward the assembly. At 956 ps, the system forms the target structure for the first time, where the linear chain shown is actually a ring under periodic boundary conditions. See Movies S7 and S8 in the SI for the equilibrium and nonequilibrium realizations for one state, respectively, and Movie S9 for nonequilibrium realizations for the two-state system.

The MC results provide a clear, idealized understanding of this relationship between the external driving forces and the assembly kinetics of patchy particle systems, while the MD simulations offer a more realistic, albeit complex, perspective. Despite the variability in the MD data, the overarching trend aligns with the MC findings, supporting the notion that external drives can be a powerful tool to rapidly steer systems toward desired configurations.

## Conclusion

Our study provides comprehensive insights into the self-assembly mechanisms of 8-patchy particle systems, employing both equilibrium and nonequilibrium conditions. The simulations have unveiled the pivotal role of external driving forces in navigating the intricate landscape of self-assembly. We have effectively modeled a process that mimics dissipative self-assembly, wherein the external drive facilitates assembly while dissipating energy, emulating the transient nature of energy consumption in biological processes. Our findings reveal an interdependence between the time to first assembly and stability in these systems, particularly pronounced under nonequilibrium conditions. This interplay showcases the potential of external forces to guide assembly processes beyond the equilibrium constraints.

We have provided comprehensive insights into the self-assembly of one-state and two-state patchy particle systems, employing both equilibrium and nonequilibrium conditions to understand the dynamics of assembly. By carrying out equilibrium simulations, we first highlighted the inherent trade-offs, where increased stability resulting from stronger interactions, comes at the price of slower assembly times. Introducing an external drive, on the other hand, circumvented these limitations in both the one-state and two-state systems by expediting the assembly process and increasing target stability. Through meticulous analysis of an order parameter that serves as a proxy for the number of formed bonds, and the system’s total entropy production, both in the absence and presence of an external driving force, we have demonstrated the influence of the external drive in directing the system toward the target structure, circumventing the barriers encountered in an equilibrium scenario. Specifically, the one-state system exhibited a smooth transition to the target structure, while the two-state system navigated a more complex energetic landscape. The observation is noticeable in the initially rougher median profiles of *R* and *S* until the target structure is assembled in the two-state system, in contrast to the smoother profile seen in the one-state system. Further, We have simulated self-assembly in crowded environments in both MC and MD, offering a closer approximation to biological scenarios and illuminating the “crowding effect” in the processes. In this pursuit, we have demonstrated an approach for introducing an external drive in the form of a time-dependent square wave affecting the patch interaction potential into MD simulations, showing the effect of the nonequilibrium conditions on reducing the assembly time, in agreement with MC simulation outcomes. Furthermore, we demonstrated the versatility of our approach by assembling both small systems of 8 patchy particles into rings and large systems of 1000 patchy particles into a mix of rings and chains, accompanied by a detailed analysis of the number and size distribution of the formed rings.

Our work contributes to the theoretical understanding of particle self-assembly, demonstrating the utility of external forces in steering assembly processes. These insights are invaluable for the controlled design of advanced materials and nanotechnological devices, where precise manipulation of assembly dynamics is crucial. While our current study provides initial qualitative insights into the entropy productions and configurational phase space by showing the order parameter *R*, we acknowledge the need for further analyses for a comprehensive understanding of the energy landscapes in future work. Looking forward, this work lays the groundwork for further exploration of nonequilibrium conditions in complex systems, opening avenues for novel methods to manipulate assembly processes across diverse scientific and industrial applications.

## Supporting Information Available

Details on the computational approach of the MC and MD simulations, as well as details on the assembly mechanism and dynamics. **Figure S1**: Schematic of two interacting patchy particles; **Figure S2**: Effects of driving forces on patchy particle assembly; **Figure S3**: Equilibrium and nonequilibrium results at high temperature; **Figure S4**: Equilibrium and nonequilibrium results in the presence of PBC. **Figures S5**: Equilibrium values of Order Parameter (*R*) and Entropy Production (*S*); **Figure S6**: Median comparison of *R* and *S* for one- and two-state systems; **Figure S7**: Median system energy for one- and two-state scenarios; **Figure S8**: Equilibrium results of 1000 patchy particles with one-state and two-states; **Figure S9**: Ring size distribution from Equilibrium simulation of 1000 patchy particles with one-state and two-states; **Figure S10**: Nonequilibrium results of 1000 patchy particles at different external drive values; **Figure S11**: Ring size distribution from Nonequilibrium simulation of 1000 patchy particles with one-state and two-states; **Figure S12**: Nonequilibrium results of 1000 patchy particles as a function of MC steps at different external drive values; **Figure S13**: Snapshot of 1000 patchy particle simulations with two-states; **Figure S14**: Interaction potential energy function between patches with high and low-energy phases; **Figure S15**: *T*_*fas*_ as a function of the square wave amplitude with a periodicity of 40 ps, in MD simulations. **Figure S16**: MC and MD simulations of Crowd system for two-state; **Figure S17**: Mechanism of external square-wave potential on bond formation (PDF)
**Movie S1**: Equilibrium MC, 8 particles, one-state (MP4)
**Movie S2**: Equilibrium MC, 8 particles, two-state (MP4)
**Movie S3**: Nonequilibrium MC, 8 particles, one-state (MP4)
**Movie S4**: Nonequilibrium MC, 8 particles, two-state (MP4)
**Movie S5**: Equilibrium MC, 1000 particles, two-state (MP4)
**Movie S6**: Nonequilibrium MC, 1000 particles, two-state (MP4)
**Movie S7**: Equilibrium MD, 8 particles with 48 crowds of one-state (MP4)
**Movie S8**: Nonequilibrium MD, 8 particles with 48 crowds of one-state (MP4)
**Movie S9**: Nonequilibrium MD, 8 particles with 48 crowds of two-state (MP4)
