# Supplementary material for: Driven Self-Assembly of Patchy Particles Overcoming Equilibrium Limitations
Source: J Chem Theory Comput. 2024 Sep 10;20(18):7700–7. doi: 10.1021/acs.jctc.4c01118 (PMC11428128; doi:10.1021/acs.jctc.4c01118)
Supplement: Supplementary file 1 — ct4c01118_si_001.pdf [file ct4c01118_si_001.pdf]

Supporting Information: Driven Self-Assembly of Patchy Particles Overcoming Equilibrium Limitations

Shubhadeep Nag[†] and Gili Bisker[*,†,‡,¶,§,‖]

†*Department of Biomedical Engineering, Faculty of Engineering, Tel Aviv University, Tel Aviv 69978, Israel*

‡*The Center for Physics and Chemistry of Living Systems, Tel Aviv University, Tel Aviv 6997801, Israel*

¶*The Center for Nanoscience and Nanotechnology, Tel Aviv University, Tel Aviv 6997801, Israel*

§*The Center for Light-Matter Interaction, Tel Aviv University, Tel Aviv 6997801, Israel*

‖*The Center for Computational Molecular and Materials Science, Tel Aviv University, Tel Aviv 6997801, Israel*

E-mail: bisker@tauex.tau.ac.il

S1 Computational Details

We focus on the self-assembly of 8 patchy particles with a target structure of 8-sided polygon or ring, with two distinct sets of simulations aimed at assessing the fast assembly time T_{fas} starting from random configuration, and the time the system remains at the target structure T_{stable} starting at the target assembly as initial conditions.

T_{fas} elucidates the kinetic efficiency of the assembly process, indicating the speed at which a disordered system transitions into an organized state under varying patch interaction energy ϵ_{patch} values. This metric sheds light on the energy barriers and assembly pathways encountered during the process. In contrast, T_{stable} evaluates the durability and energetics of the assembled structure over time, thus serving as an indicator of its stability and energetic favorability.

To ensure robust statistics of these two parameters, we conducted 20 independent MC simulations for each ϵ_{patch} value, varying the seed of the random number generator. Additionally, for T_{fas} analysis, each simulation commenced from a random starting configuration. Collectively, T_{fas} and T_{stable} provide a comprehensive framework for assessing the assembly dynamics and stability of the resulting structures. All these simulations were conducted within a cubic simulation cell with dimensions of $20 \times 20 \times 20$ Å^3 to gain insights into the self-assembly mechanism. Subsequently, for patchy particle simulations involving crowding agents, we used a smaller cubic simulation cell with a side length of $10 \times 10 \times 10$ Å^3. This choice is motivated by the desire to increase hindrance to the patchy particles during target structure formation, mimicking densely packed systems.

Both MC and MD simulations were employed to study the assembly of the patchy particles into an 8-sided polygon, both in the absence and presence of an external drive, which is incorporated to enhance the self-assembly process. In MC, the drive modulates the energy of configurations based on the proximity of nearest neighbor particles, reducing energy for favorable assemblies and increasing it for unfavorable configurations, as detailed in Section S1.2. This asymmetry breaks detailed balance, shifting the system dynamics into a nonequi-

librium regime and resulting in Dissipative Self-Assembly (DSA). In MD, where direct energy manipulation is not viable, we switch between two potential energy levels differing by a constant value, manipulating transition rates between particle states, as detailed in Section S1.3. Lower potential promotes the formation of nearest neighbor pairs, while higher potential hinders their separation. These asymmetric transition rates favor assembly while breaking detailed balance. Both methods used for our MC and MD simulations employ the nonequilibrium force to direct the system from random behavior toward targeted structure formation.

S1.1 Modeling Patchy Particles

We conceptualized each bead (the core of a patchy particle) and its associated patches as a single rigid body, employing the Kern-Frenkel[1–5] approach to model this system. The total interaction potential of a patchy particle, $U_{particle}$, is divided into two components, the bead potential, denoted as U_{bead}, and the patch potential, referred to as U_{patch}:

$$U_{particle} = U_{bead} + U_{patch} \tag{S1}$$

Here, the beads are modeled with the repulsive part of the 6-12 Lennard-Jones potential and patches with both square well potential and an angular potential. Further, the patch potential is different for identical-state systems and two-state systems. Uniform-state systems patch potential is denoted as $U^{\alpha\alpha}_{patch}$ and two-state system as $U^{\alpha\beta}_{patch}$.

The expressions for U_{bead} is expressed as:

$$U_{bead} = 4\epsilon_{bead}\left(\frac{\sigma_{bead}}{r_{ij}}\right)^{12} \tag{S2}$$

where σ_{bead} denotes the diameter or vdW radii, ϵ_{bead} denotes the strength of the potential of the bead, and r_{ij} denotes the distance between i^{th} and j^{th} particles. The diameter and the depth of the bead potential are set to be 2 Å and 0.75 kJ/mol, respectively, and they are

kept constant for particles of both the one-state and two-state systems. For all particles, the angle between the patches with respect to the core of the specific particle is set at 135 deg to ensure the formed structure is an octagon.

The expression of patchy potentials $U^{\alpha\alpha}_{patch}$ and $U^{\alpha\beta}_{patch}$ are:

$$\begin{aligned} U^{\alpha\alpha}_{patch} &= \epsilon_{patch} \sum_{\alpha=1}^{2} f(\vec{r}_{ij}, \hat{n}^{\alpha}_{i}, \hat{n}^{\alpha}_{j}), \quad if \quad \sigma_{bead} \leq |\vec{r}_{ij}| < (\sigma_{bead} + \delta) \\ U^{\alpha\beta}_{patch} &= \epsilon_{patch} \sum_{\alpha,\beta=1}^{2} f(\vec{r}_{ij}, \hat{n}^{\alpha}_{i}, \hat{n}^{\beta}_{j}), \quad if \quad \sigma_{bead} \leq |\vec{r}_{ij}| < (\sigma_{bead} + \delta) \end{aligned} \tag{S3}$$

where ϵ_{patch} is the interaction potential between patches, $\delta/2$ is the length of the patch, $f(\vec{r}_{ij}, \hat{n}^{\alpha}_{i}, \hat{n}^{\alpha}_{j})$ and $f(\vec{r}_{ij}, \hat{n}^{\alpha}_{i}, \hat{n}^{\beta}_{j})$ denote the angular interaction potential for the 1 state and two states systems, $\vec{r}_{ij}$ is the vector connecting the centers of the ith and j^{th} particles, and $\hat{n}^{\alpha/\beta}_{j}$ denotes the unit vector connecting the center of the bead and the center of patches of the patchy particle of state α or β, respectively. This potential between the patches is effective only when the distance between two particles lies within the range of σ_{bead} and $\sigma_{bead}+\delta$.

The expressions for the angular interaction potentials are:

$$f(\vec{r}_{ij}, \hat{n}^{\alpha}_{i}, \hat{n}^{\alpha}_{j}) = \begin{cases} 1, & if \quad \vec{r}_{ij}.\hat{n}^{\alpha}_{j} \geq \cos(\theta^{max}) \\ 0, & otherwise \end{cases} \tag{S4}$$

and:

$$f(\vec{r}_{ij}, \hat{n}^{\alpha}_{i}, \hat{n}^{\beta}_{j}) = \begin{cases} 1, & if \quad \vec{r}_{ij}.\hat{n}^{\alpha}_{j} \geq \cos(\theta^{max}) \ \& \ \vec{r}_{ji}.\hat{n}^{\beta}_{j} \geq \cos(\theta^{max}) \\ 0, & otherwise \end{cases} \tag{S5}$$

where θ^{max} is half of the angular width of the patch, and it is maintained at a particular value to ensure a single bond per patch. This angle is, therefore, related to the vdW radius of the bead (σ_{bead}) and the length ($\delta/2$) of the patches in the following manner:[6,7]

$$\sin(\theta^{max}) \leq \frac{\sigma_{bead}/2}{\sigma_{bead} + \delta} \tag{S6}$$

A schematic presentation of two patchy particles separated by a distance r_{ij}, with all the parameters defined in the text, is shown in Fig. S1.

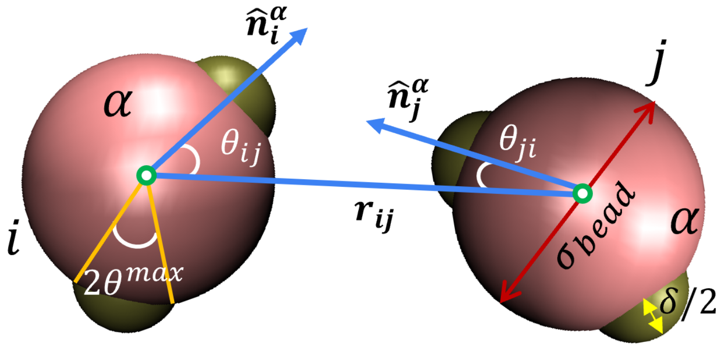

Figure S1: Schematic presentation of two patchy particles, with all the parameters defined in the text.

The length of the patches in this work is maintained at a value of 0.425 Å which made the θ^{max} value set at 0.35 radian according to Eq. S6. This guarantees that the interaction potential between patches is active only when the angle (say, θ_{ij}) formed by a patch's unit vector, $\hat{n}_i^\alpha$ of the ith particle, and the vector connecting between the centers of the two particles (i and j), $\vec{r}_{ij}$, is less than or equal to half of the patch's angular width, θ_{max}, thereby restricting each patch to form a single bond with another patch.

The latter distance refers to the minimum distance between the centers of two patchy particles, effectively connecting both particles' patches. This specific condition is not only crucial for the formation of a single bond per patch but is also essential for assembling a ring from the 8 particles. Therefore, in the two-state system, patches of patchy particles with different states attract each other, while patches with the same internal state exhibit no interaction. For the single-state system, since all particles share the same internal state, patches of any two particles interact.

S1.2 Monte Carlo Simulations

We employed MC simulations to study patchy particle systems under both equilibrium and nonequilibrium conditions, the latter involving an external drive. These simulations were performed using a custom-developed in-house program. Every simulation was conducted at a temperature of 65 K and spanned 7×10^6 Monte Carlo steps. In these simulations, reflective walls were implemented along all the cartesian axes. Each simulation cycle encompassed two fundamental processes, namely, the translation of beads and the rotation of patches. The choice of 7×10^6 MC steps for our simulations was carefully considered and validated through extensive testing, such that the observed behaviors and trends remained consistent for longer simulations. Furthermore, in this simulation length, the motion of particles can be mapped to a diffusive process, where 7×10^6 MC steps correspond to approximately $10^2 - 10^3$ diffusion times, confirming the adequacy of the simulation length for our study.

In the equilibrium Monte Carlo simulations, we calculated the total interaction energy both before and after executing the translation and rotation processes. Subsequently, we employed the Metropolis scheme to assess each attempted move, determining whether it should be accepted or rejected.[8]

To overcome the equilibrium trade-off, an external drive was applied between patches to drive the system into a nonequilibrium state:

(1) When patches from two distinct particles, which are nearest neighbors in the target structure, come into proximity during a subsequent Monte Carlo step, an external drive ($-\epsilon_{drive}$) is applied to their total interaction energy ($U^{new}_{Particle}$). This effectively reduces the total energy in the new configuration, thus promoting the formation of a virtual bond. The determination of whether to accept or reject this interaction is made by computing the acceptance probability w:

$$w = min\big[1, exp\big\{ - (U^{new}_{Particle} - \epsilon_{drive} - U^{old}_{Particle})/k_B T\big\}\big] \tag{S7}$$

here, $U^{new}_{Particle}$ is the energy of the particle at the proposed MC step while $U^{old}_{Particle}$ denotes the energy of the particle in the current position. The term k_BT represents the Boltzmann factor, where k_B stands for the Boltzmann constant, and T represents the temperature.

(2) Correspondingly, if two patches, which were neighbors in the target structure, move apart in a subsequent MC step, an external drive $(+\epsilon_{drive})$ is applied. This operation increases the total interaction energy in the proposed step, $U^{new}_{Particle}$, and unfavour the bond breaking. The decision to accept or reject this interaction is determined by computing:

$$w = min\big[1, exp\big\{-(U^{new}_{Particle} + \epsilon_{drive} - U^{old}_{Particle})/k_BT\big\}\big] \quad \text{(S8)}$$

(3) If a virtual bond exists between two patches that are neighbors to each other in accordance with the target structure and persists in the next MC step, the patchy interaction remains unchanged as no external drive is added or subtracted. Therefore, the acceptance or rejection in this scenario is determined by the Metropolis scheme only given below:

$$w = min\big[1, exp\big\{-(U^{new}_{Particle} - U^{old}_{Particle})/k_BT\big\}\big] \quad \text{(S9)}$$

A schematic figure of how the external drive acts between patches of different particles is shown in Fig. S2. In this figure, we only used same-state particles. However, for the dual-state system, the drive works similarly between different states. This external drive essentially breaks the detailed balance and drives the system out of equilibrium. Thus, this model relies on energy from external sources to form and maintain the target structure as it is done in the assembly of tubulin dimers or G-actin in the formation of microtubules or actin filaments through GTPase or ATPase hydrolysis.

To enhance the accuracy of simulating biological events, our study employs a minimalistic model of patchy particles, designed to emulate cytoskeletal polymers. By simulating a larger number of particles, along with additional cellular components, we aim to replicate complex biological systems more faithfully. In this simulation setup, we include the same 8 patchy

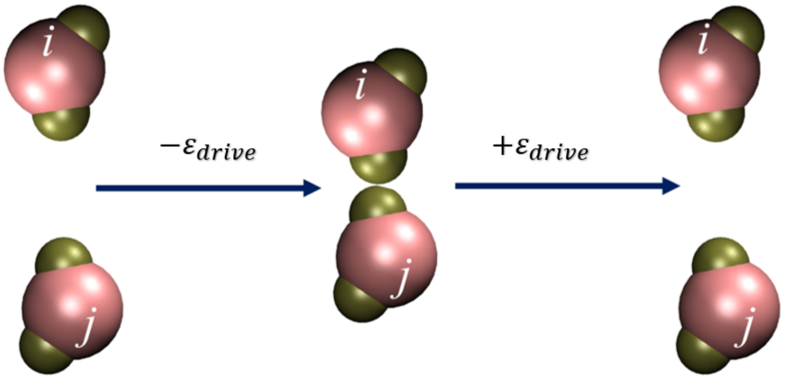

Figure S2: The effect of the drive on the assembly protocol of patchy particles utilized in this study. The interactions between patches of adjacent particles, as stipulated by the target structure, are modulated by an external drive, ϵ_{drive}. When particles approach each other, ϵ_{drive} is deducted from the total energy, facilitating bond formation by making it thermodynamically favorable. Conversely, when particles diverge, ϵ_{drive} is added to the total energy, rendering bond dissociation thermodynamically less favorable.[9]

particles and 48 crowding agents. These crowding agents interact with each other and the patchy particles via Eq. S2 characterized by a parameter σ_{crowd} of 2 Å and a potential depth of 1 kJ/mol. The simulation is conducted in a cubic cell with dimensions of 10 Å, at a temperature of 65 K, and runs for 7×10^6 MC steps. During this simulation of patchy particles with crowding agents, periodic boundary conditions (PBC) are implemented in all Cartesian directions. Adhering to the rule that each patch can form only a single bond, we anticipate the formation of various structures in this setup. The simulation reaches the target structure once the first 8-ring structure is successfully assembled.

We have also simulated a larger system consisting of 1000 particles in a simulation box with dimensions of $100 \times 100 \times 100$ Å^3 to maintain a number density of 0.001 Å^{-3}, consistent with our previous simulation of 8 patchy particles in a cubic simulation box with a side length of 20 Å, using either one or two states. The walls of this simulation cell were modeled with PBC. During the simulation, we maintained the neighbor list with a cutoff distance of 20 Å and updated this list every 10 MC steps. The simulations were conducted for 5×10^6 MC steps. All other simulation parameters, including those for the patches, were kept identical to those used in the 8-patchy particle simulation.

In essence, for an 8-particle system with 1 state, the drive is applied indiscriminately since any two particles can be nearest neighbors. However, in systems with two distinct states of α and β, the drive is selectively applied to α-β particle pairs that constitute the nearest neighbors in the target structure. This tailored application of the external drive ensures the directed assembly of the desired structure without compromising the thermodynamic principles that govern nonequilibrium processes.

S1.3 Molecular Dynamics Simulations

To support and complement the results of the Monte Carlo simulation, we performed Molecular Dynamics (MD) simulations of the same system mentioned above in both equilibrium and nonequilibrium conditions. The MD simulations have been performed in NVT ensemble for 8 ns with an integration timestep of 0.2 fs in the canonical ensemble with the velocity Verlet algorithm[10] using Large-scale Atomic/Molecular Massively Parallel Simulator (LAMMPS)[11] at 40 K. The simulation temperature was regulated using the Nose-Hoover thermostat,[12,13] employing a coupling constant of 200 fs. This setup effectively maintained the target temperature at 40 K, with a permissible fluctuation margin of ±10 K. The temperature value in the MD simulations was set to a lower value than in the MC simulations, to counteract the significant temperature fluctuations often observed in MD simulations at higher temperatures.

While the temperatures chosen for the MC and MD simulations are different, this choice was driven by the distinct nature of the two simulation methods. MD simulations conducted at 65 K experienced significant temperature fluctuations, with an error margin of ±15 K, making it challenging for the system to assemble with an ϵ_{patch} value of 3.7 kJ/mol used in the MC simulations. To mitigate this issue and ensure stable simulation conditions, we set the MD simulation temperature to 40 K. This temperature setting allowed us to achieve more relevant and less noisy results. For higher ϵ_{patch} values, a temperature of 65 K would become adequate, as the Boltzmann factor relationship dictates that increasing the temperature

requires a corresponding increase in interaction energy.

The bead of the patchy particle is modeled with pseudo hard-sphere potential taken from the work of Jover *et al.*:[14]

$$U_{bead} = \begin{cases} \lambda_r \left(\frac{\lambda_r}{\lambda_a}\right)^{\lambda_a} \epsilon_r \left[\left(\frac{\sigma_{bead}}{r}\right)^{\lambda_r} - \left(\frac{\sigma_{bead}}{r}\right)^{\lambda_a}\right] + \epsilon_r, & \text{if } r < \left(\frac{\lambda_r}{\lambda_a}\right)\sigma_{bead} \\ 0, & \text{if } r \geq \left(\frac{\lambda_r}{\lambda_a}\right)\sigma_{bead} \end{cases} \tag{S10}$$

here, the coefficients for attractive and repulsive forces are denoted by $\lambda_a = 49$ and $\lambda_r = 50$, respectively. The parameter ϵ_r represents the interaction strength in the pseudo hard-sphere (PHS) model, characterizing the energy associated with the interactions. Additionally, σ_{bead} corresponds to the van der Waals radius of the patches on the particles. The variable r signifies the distance measured from the center of one particle to another. Further, the values for ϵ_r and σ_{bead} are set at 0.24 kJ/mol and 2 Å, respectively. These choices are aligned with the parameters utilized by Espinosa *et al.* in their investigation of liquid-vapor coexistence in systems comprising patchy particles.[15] We maintain the k_BT/ϵ_r ratio at 1.38 for T = 40 K in choosing ϵ_r, ensuring an accurate representation of the particles as pseudo-hard spheres, consistent with the modeling approach by Jover *et al.*[14] These parameters have been established to provide a realistic representation of particle interactions within the simulated environment.

The interaction energy between patches of different particles, U^{csw}_{patch}, was modeled using a continuous attractive square-well potential:[16]

$$U^{csw}_{patch} = -\frac{1}{2}\epsilon_{patch}\left[1 - \tanh\left(\frac{r - r_w}{\alpha}\right)\right] \tag{S11}$$

where r is the distance between the centers of two patches on different particles, r_w is the radius of the attractive well, and α dictates the steepness of the well. In our simulations, r_w corresponds to the length of the patches that we have defined. The mass of the bead is taken as 10 amu and the patches as 1 amu. However, the relative difference in the mass of both

bead and patch does not affect the system dynamics because our external drive protocol operates independently of inertia.

The value of ϵ_{patch} is taken to be 3.7 kJ/mol. This particular value of ϵ_{patch} was selected to highlight the effectiveness of an external drive in facilitating the assembly process, as it represents a regime where spontaneous assembly is difficult to achieve under equilibrium conditions for a set of parameters in both MC and MD. To restrict the formation to one bond per patch, we have selected values for α and r_w as 0.01 Å (equal to $0.005\sigma_{bead}$) and 0.24 Å (equal to $0.12\sigma_{bead}$), respectively, as taken by Espinosa *et al.* in their work.[15]

The mass of the crowding agent is taken to be 2 amu with a vdW radius of 2 Å. The rationale behind choosing the same diameter for the patchy particles and crowd agents is to generalize the crowded environment observed in various systems. For example, in cellular environments, macromolecules like proteins, nucleic acids, and small metabolites often occupy similar spatial dimensions, creating a densely packed milieu. Additionally, certain solute-solvent systems also exhibit this characteristic, such as small gold clusters in a neon (Ne) noble gas matrix. In this system, the gold dimer/trimer has a relative size of approximately $0.25 - 0.3$ nm, while the diameter of Ne is about 0.27 nm.[17] These examples illustrate that using particles of the same size can accurately reflect the complexity and crowdedness of natural environments. Future research could vary the relative sizes and masses of patchy particles and crowding agents to gain further insights into self-assembly processes in different environments. The crowds interact with the beads of the particle with the same interaction present between two beads (see Eq. S10). The trajectory and energy between patches are stored every 100 fs throughout the simulation for further analysis.

To carry out nonequilibrium MD simulation, we introduce a time-dependent square wave potential in addition to the patchy particle interaction. Incorporating the external drive in the form of a periodic potential is inspired by both biological phenomena and experimental studies, mirroring mechanisms such as voltage-gated ion channels in neurons[18] and bistable genetic switches in cellular functions,[19] which undergo transitions in response to external

stimuli. Experimental validations of similar concepts of using time-dependent periodic potential have been demonstrated, such as the directed assembly of non-spherical particles under electromagnetic fields,[20] and the control of particle assembly dynamics.[21] These studies lend empirical support to our simulation-based exploration of self-assembly and structural stability under nonequilibrium conditions, highlighting the significant potential of external forces in guiding assembly processes beyond equilibrium constraints.

Our simulation approach, which utilizes the LAMMPS package, involves using the square wave potential to perturb the system with a momentary change in force, encouraging bond formation between adjacent particles. We modulate the high energy and low energy phase of the square wave potential around the base interaction potential of the patchy particles (U^{csw}_{patch}) at half the frequency of the applied square wave, transitioning to nonequlibrium conditions. The baseline potential at 3.7 kJ/mol, and the high and low energy phase potentials of the applied square wave, are depicted in Fig. S14. Although the relative difference in the mass of the bead and patch affects T_{fas} and T_{stable}, it does not influence the application of our design principle. This is because the time-dependent square potential we employ is mass-independent, ensuring that both equilibrium and nonequilibrium scenarios are uniformly affected. Our simultaneous work delves into this technique, providing comprehensive details on its conception, implementation, and effectiveness in establishing nonequilibrium conditions in MD simulations.[22]

S1.4 Physical Origin of External Driving Force

The physical origin of the external drive is inspired by natural biological systems where energy is constantly consumed to drive assembly processes out-of-equilibrium, similar to how molecular motors and the hydrolysis of GTP and ATP drive the polymerization of microtubules and actin filaments. During polymerization, the hydrolysis of GTP to GDP in tubulin and ATP to ADP in actin monomers leads to conformational changes that support the dynamic polymer structures.[23,24] In real systems, such forces could be implemented

through magnetic or electric fields,[20,21] optically induced forces,[25] or chemical potential gradients.[26] Unlike a simple change in temperature, which affects the kinetic energy of all particles uniformly, these externally applied drives are specifically designed to modulate the interaction energies between patches on adjacent particles. This targeted influence is essential for selectively promoting bond formation and inhibiting bond dissociation. These drives, as a result, inherently break the detailed balance, a process that is necessary for replicating the nonequilibrium behavior observed in biological systems, where continuous energy input overcomes equilibrium limitations to achieve order. This approach not only advances the understanding of nonequilibrium self-assembly processes but also offers new strategies for designing materials with novel functionalities.

S2 High-Temperature Dynamics of Patchy Particles

In this section, we explore the dynamics of an 8 patchy particle system with one internal state at a biologically relevant temperature of 300 K. The motivation for this analysis stems from the need to generalize the effectiveness of an external drive on the self-assembly design under the temperature that closely resemble those found in natural and experimental settings. By conducting simulations at 300 K, we aim to provide a comprehensive understanding of how temperature influences the stability and kinetics of patchy particle assemblies. This section presents detailed results, comparing the formation and stability of the target structures across different interaction strengths and external biases, thereby offering deeper insights into the nonequilibrium self-assembly dynamics.

We first carried out equilibrium MC simulations over a range of ϵ_{patch} values, from 0 to 400 kJ/mol, to investigate the temperature dependence of the self-assembly process. For each value of ϵ_{patch}, we quantified the T_{fas}, and the T_{stable}, in MC steps, as shown in Figs. S3(a) and S3(b), respectively. At low interaction energies (ϵ_{patch} ranging from 0 to 40 kJ/mol), the median T_{fas} remains relatively constant at the simulation length (7×10^6 MC steps),

indicating that the particles do not form a stable assembly within the allotted simulation time. As ϵ_{patch} increases from $\sim$ 45 kJ to higher values, we observe a notable decrease in T_{fas} which eventually stabilizes with a further increase in ϵ_{patch}.

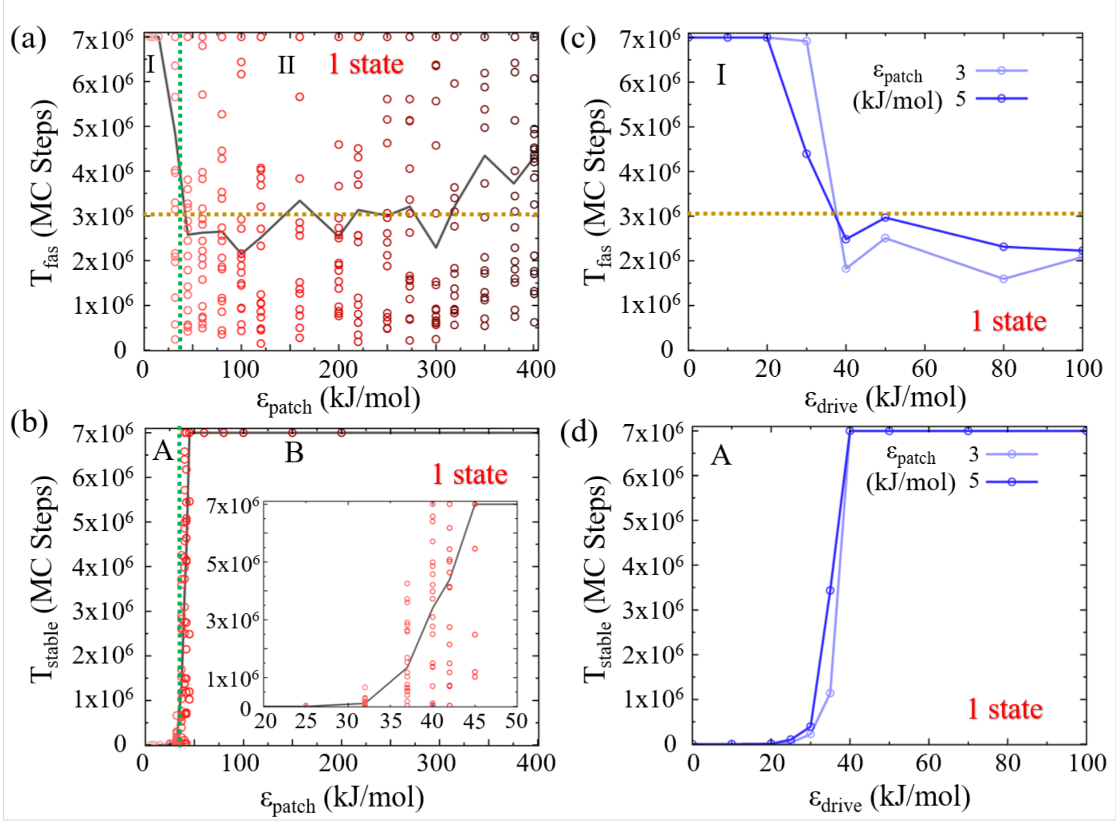

Figure S3: Dynamics of 8 patchy particle systems at 300 K. Equilibrium Simulation: (a) Median T_{fas} values (black line), and (b) Median T_{stable} values alongside individual respective simulation results (red circles) for various ϵ_{patch} values. The golden dotted line denotes the average of the median values in Region II. Nonequilibrium Simulation: (c) The effect of external bias between the patches on the variation of T_{fas} and (d) T_{stable} for ϵ_{patch} values in regions I and A, respectively. Each value in (c) and (d) is the median of 20 distinct simulations.

Although the initial variation in T_{fas} with increasing patch interaction is similar to the observations from the equilibrium simulation at 65 K (see Fig. 2(a) and 2(c)), the region III observed at 65 K at higher patch interaction energy is not present at 300 K. Therefore, we consider the entire range as region II in this case and obtain the optimal value by averaging the median values over all these energy levels, yielding $\sim 3 \times 10^6$ MC steps.

The absence of Region III may occur because the higher temperature elevates the sys-

tem's energy, allowing it to overcome kinetic traps that would otherwise be present at lower temperatures relative to the system's interaction energy. The higher temperature of 300 K appears to impede the formation of these kinetic traps, thereby moderating the impact of strong interactions. This observation is consistent with previous studies showing that higher temperatures can provide sufficient thermal energy to overcome kinetic barriers, facilitating transitions out of kinetic traps.[27]

Regarding the equilibrium result at 300 K for examining the target stability (see Fig. S3(b)), we observed a similar result to that at 65 K. For low ϵ_{patch} values, all systems display relatively low T_{stable} values (Region A), indicating insufficient stability of the target structures. As ϵ_{patch} increases beyond $\sim$ 35 kJ/mol, T_{stable} rises, approaching the simulation's maximum duration (Region B), with structures remaining assembled throughout.

From the nonequilibrium simulation (see Fig. S3(c)), we observed that for ϵ_{patch} of 3 kJ/mol and 5 kJ/mol of the region I, T_{fas} remains at the maximum simulation length until ϵ_{drive} exceeds 25 kJ/mol, indicating the necessity of an adequate driving force for assembly. When ϵ_{drive} reaches $40-50$kJ/mol, T_{fas} significantly reduces and stabilizes around the equilibrium value of Region II (3×10^6 MC steps). The external drive also enhances target stability, as shown by the higher T_{stable} for increasing drive values for the selected ϵ_{patch} values of Region A (see Fig. S3(d)), consistent with previous findings at 65 K.

From the results at 300 K, it is noteworthy that the threshold ϵ_{patch} value required for stable assembly formation shifts from 5 kJ/mol at 65 K to approximately $35-40$ kJ/mol at 300 K. Similar shifts have been observed in the value of the external drive for a given interaction value between patches. This increase in interaction energy is necessary to facilitate the assembly formation and its stability, which arises due to the relative value of the higher temperature compared to the previously simulated temperature. This suggests that our key findings are robust and can be extrapolated to higher temperatures. The Boltzmann factor ($e^{-\frac{E}{k_B T}}$) plays a crucial role here, indicating that the interaction strength is needed to overcome kinetic barriers scales with temperature. Consequently, the assembly process

remains governed by the same underlying principles, even at higher temperatures.

S3 Effect of boundary wall on Patchy particle self-assembly

In our previous MC simulations, we employed reflective boundary conditions to facilitate a clear visualization of the ring formation process of eight patchy particles in both one and two-state systems. We recognize that using such a wall may lead to more frequent particle collisions, which could potentially accelerate the assembly rate compared to PBC. To ensure that this does not introduce any artifacts into our key findings, we conducted additional simulations using PBC. Our aim was to validate our design principle of facilitating assembly and confirm that the observed trends in T_{fas} and T_{stable} are robust regardless of the boundary conditions used. These additional simulations are performed on an 8-patchy particle system with two states using the exact same simulation parameters employed for reflective boundary conditions (Fig. S4).

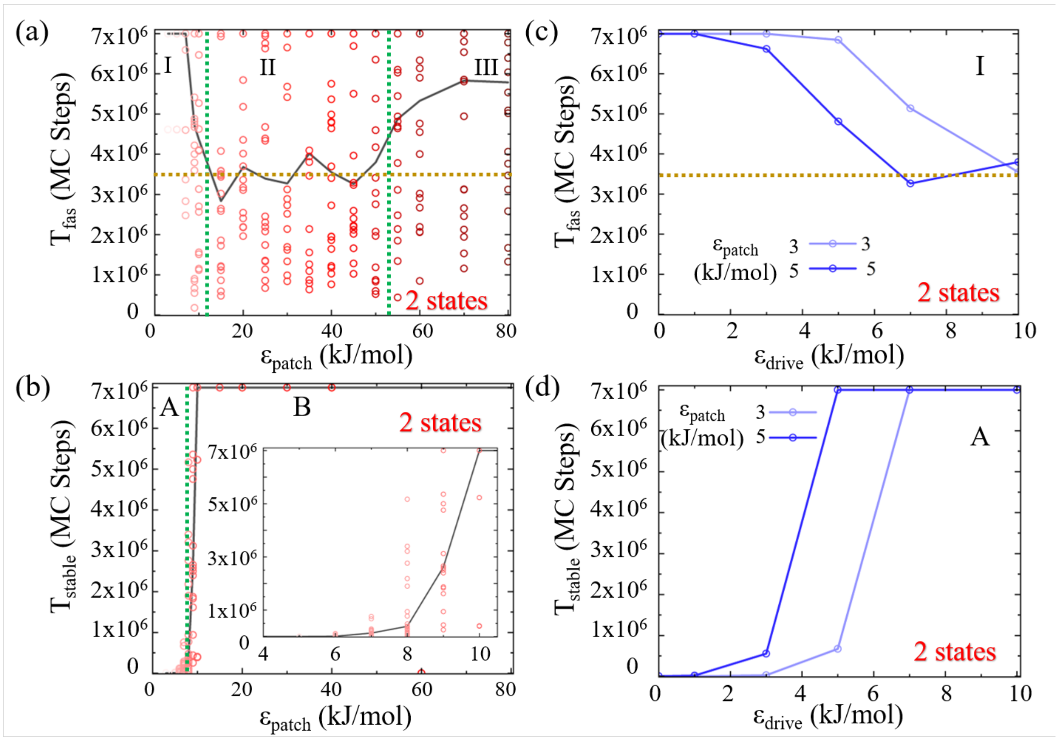

Figure S4: MC simulation results over 20 distinct simulations of 8 patchy particles with 2 internal states in the presence of PBC. (a) Median T_{fas} values (black line) across different ϵ_{patch} from equilibrium simulation. The ϵ_{patch} range is segmented into three regions (green dotted lines), with the average T_{fas} over the median values in the middle Region II depicted by a golden dotted line. (b) Median T_{stable} values across different ϵ_{patch} from equilibrium simulation. The ϵ_{patch} range is categorized into two distinctive zones (green dotted lines). Insets: Zoom into the lower ϵ_{patch} range. Individual realizations are shown in red circles. (c) Median T_{fas} values from nonequilibrium simulations for ϵ_{patch} value of 3, and 5 kJ/mol, as a function of the external drive. The golden dotted line denotes the average value of T_{fas} in the intermediate Region II. (d) Median T_{stable} for the same ϵ_{patch}, as a function of the external drive from nonequilibrium simulations.

Fig. S4(a) shows T_{fas} as a function of ϵ_{patch}, with three distinct regions (I, II, III) marked by vertical green dashed lines, similar to what we have observed for the same system in the presence of reflective boundary conditions (see Fig. 2(b)). Fig. S4(b) plots T_{stable} against ϵ_{patch}. Here also, the trend is similar to the previous outcome (see Fig. 2(d)), dividing the range into two regions of low (Region A) and high (Region B) stability. Fig. S4(c) depicts T_{fas} versus ϵ_{drive} for ϵ_{patch} values of 3 kJ/mol and 5 kJ/mol of Region I, showing a decrease in T_{fas} with the application of ϵ_{drive}. Fig. S4(d) displays T_{stable} against ϵ_{drive} for the same ϵ_{patch}

values of Region I, and we observed similar behavior in that the presence of the external drive improves stability. Therefore, by using the PBC condition, we can confirm that the key findings in RBC are consistent.

S4 Patchy particles self-assembly mechanism

The value of the order parameter, R, which is defined as the ratio of the system's instantaneous energy between the patches to the corresponding total energy between patches in the target state, as a function of the MC steps for a one-state system at equilibrium is presented in Fig. S5(a) for a representative realization. R basically fluctuates around small values, 0, 1/8, and 2/8, underscoring the challenge of forming the target structure under such conditions. This challenge is also reflected in the behaviors of the total entropy production, S, as a function of the MC steps (Fig. S5(b)), showing fluctuations around small values. Similar fluctuations in R and S are also observed for the case of the two-state system, as seen in Fig. S5(c) and Fig. S5(d), respectively.

The pronounced fluctuations in R, evident across both system types, reflect a significant challenge in the spontaneous formation of the target assembly, a scenario that starkly contrasts the driven systems discussed in the context of Fig. 4(a) and 4(c) of the main text. The limited entropy production, interspersed with irregular peaks, correlates with the fluctuating nature of R and indicates an absence of directional progress toward system assembly. The insets in each panel in Fig. S5 magnify the initial fluctuations, providing an essential perspective on the early-stage dynamics and the resultant inability to achieve structural stability. The variance observed here is critical to understanding the thermodynamic barriers that impede the system's path to self-organization in the absence of an external drive.

Comparing these findings to the driven systems showcased in Fig. 4 of the main text, where both R and S demonstrate a rapid increase, it becomes clear that the external driving forces assume a critical role in surmounting the entropic and energetic obstacles inherent

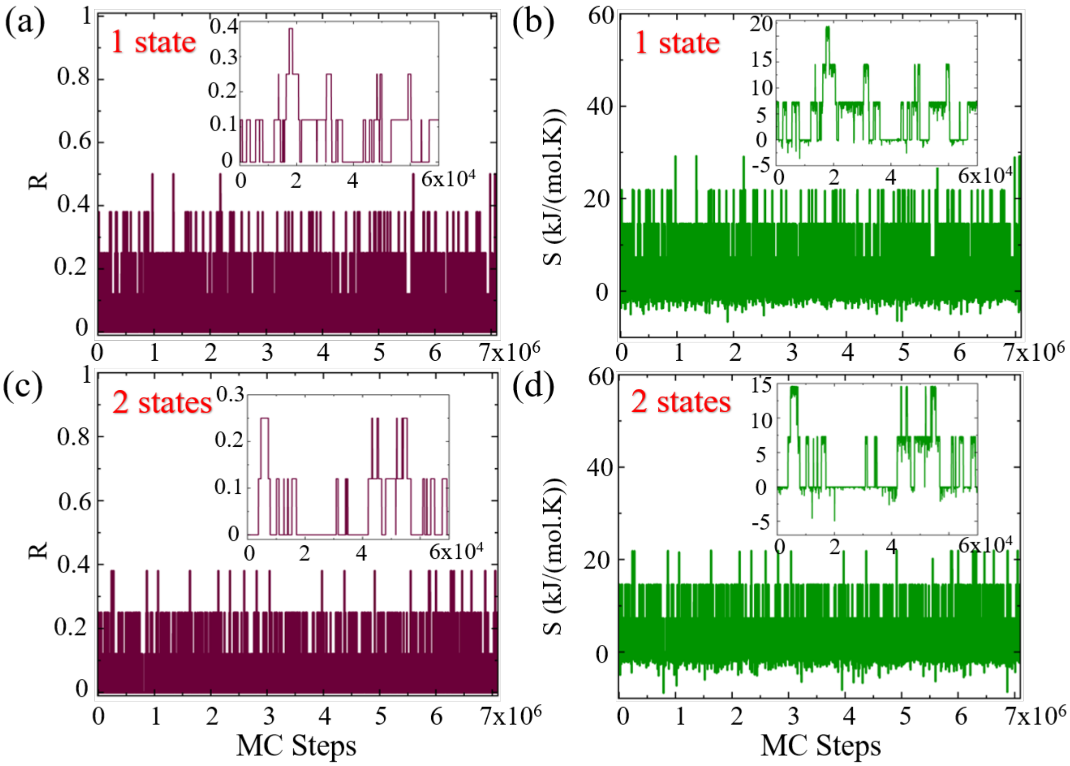

Figure S5: Representative realizations under equilibrium conditions, without an external drive. (a) The order parameter, R, as a function of MC steps for the one-state system. (b) The entropy production, S, as a function of MC steps for the one-state system. (c) The order parameter, R, as a function of MC steps for the two-state system. (d) The entropy production, S, as a function of MC steps for the two-state system. All simulations were performed for a patch interaction energy of 4 kJ/mol. Insets in each panel offer a zoomed-in view of the initial fluctuations observed in these simulations. These results are derived from one of the 20 distinct simulations, each differing in the initial configuration.

in the equilibrium state. Consequently, external driving forces are essential, providing the energy to facilitate bond formation and guiding the transition from disordered states to organized structures.

To understand the comparative dynamics of reaching the target structure for both the one-state and two-state systems in the presence of an external drive, we plotted the median of the order parameter, R, and entropy production, S, as functions of MC steps, of 20 distinct realizations, each with random initial conditions, conducted under patch energy (ϵ_{patch}) of 4 kJ/mol and a driving energy (ϵ_{drive}) of 4 kJ/mol (Fig. S6). The total entropy production, S, is obtained at every MC step from the sum of entropy produced by each particle at that

step, summing across all particles in the simulation.

The evolution of the median value of R for the one-state system, illustrated in Fig. S6(a), shows a relatively smooth transition to an ordered target, suggesting a streamlined path toward self-assembly of the encoded target structure. The value of R for the two-state system, on the other hand, exhibits significant fluctuations, indicating a more rugged energy landscape that the particles must navigate to assemble (Fig. S6(a)). The inset within the graph, showing the initial 1×10^6 MC steps, offers a detailed view of the rapid increase for both systems, with the two-state system slightly lagging in reaching the target state. Fig. S6(b) shows the rapid increase in the total entropy production for both systems, resulting from the nonequilibrium driving. Compared to the one-state system, the two-state system has lower initial entropy production, which could imply a more disordered starting configuration during the early stages of the simulation.

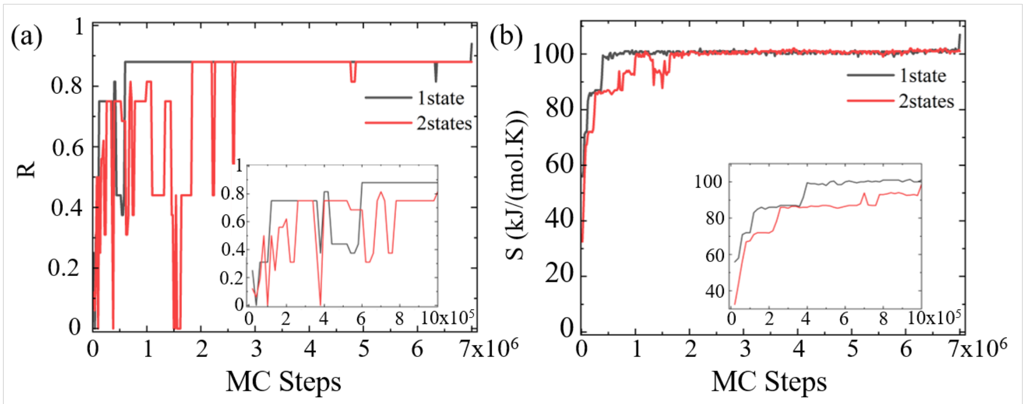

Figure S6: The variation of the median of (a) the order parameter, R, and (b) the total entropy production, S, as a function of the MC steps for the one-state system (black) and two-state system (red) calculated based on 20 distinct realizations. The simulations were conducted with a patchy interaction energy of 4 kJ/mol in the presence of an external driving force of 4 kJ/mol.

As MC steps increase, both systems reach a plateau in both R and S, indicating the formation of a stable assembly, which, in this context, is the target structure. While the one-state system follows a more efficient path, mirrored by its smoother order parameter trajectory and rapid entropy increase that suggests a simpler energy landscape, the two-state

system undergoes a more nuanced assembly process, characterized by greater fluctuations, indicative of a sequence of intermediate states and transitions, and a broader spectrum of accessible configurations.

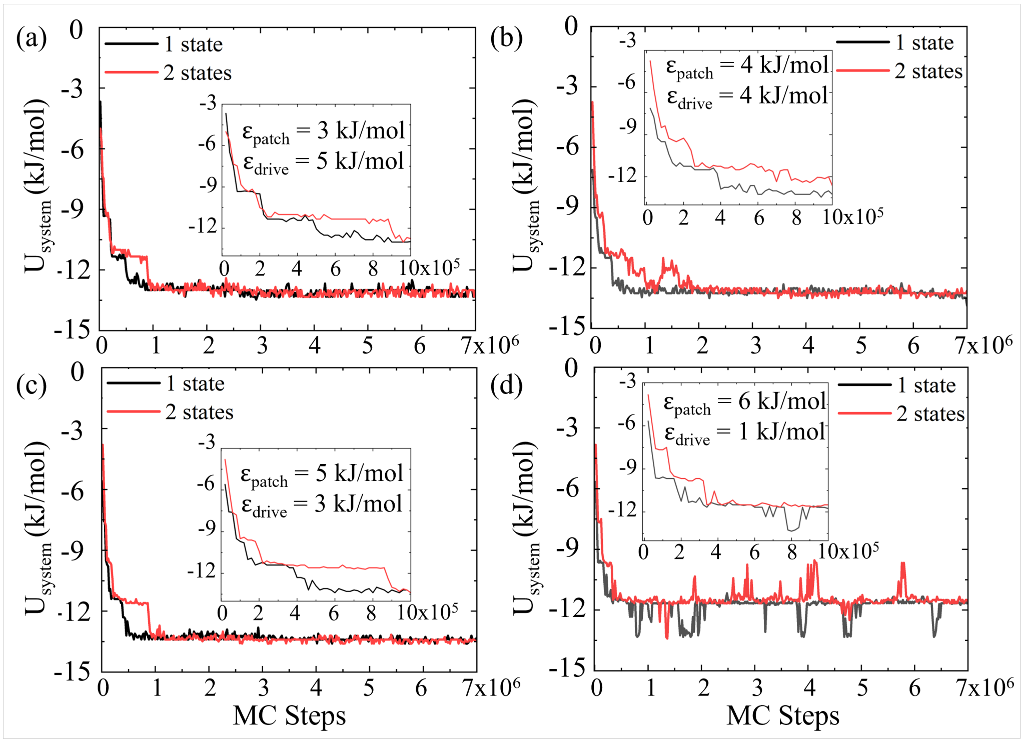

Figure S7: Median total energy of the system, U_{system}, as a function of the MC steps for the one-state (black) and two-state (red) systems under different interaction potentials and drive values. (a) $\epsilon_{patch} = 3$ kJ/mol and $\epsilon_{drive} = 5$ kJ/mol (b) $\epsilon_{patch} = 4$ kJ/mol and $\epsilon_{drive} = 4$ kJ/mol. (c) $\epsilon_{patch} = 5$ kJ/mol and $\epsilon_{drive} = 3$ kJ/mol. (d) $\epsilon_{patch} = 6$ kJ/mol and $\epsilon_{drive} = 1$ kJ/mol. The energies are normalized by the corresponding patchy interaction values.

To solidify our assertion that the one-state system approaches the target structure with faster assembly rate and fewer fluctuations compared to the two-state system, we track the system's total energy profiles for different interaction potentials, subject to varying external driving forces (Fig. S7). The respective energy trajectories are presented for $\epsilon_{patch} = 3$ kJ/mol and $\epsilon_{drive} = 5$ kJ/mol (Fig. S7(a)), $\epsilon_{patch} = 4$ kJ/mol and $\epsilon_{drive} = 4$ kJ/mol (Fig. S7(b)), $\epsilon_{patch} = 5$ kJ/mol and $\epsilon_{drive} = 3$ kJ/mol (Fig. S7(c)), and for $\epsilon_{patch} = 6$ kJ/mol and $\epsilon_{drive} = 1$ kJ/mol (Fig. S7(d)). These trajectories are derived from the median values across 20 MC simulation sets, where the insets show the initial variations.

The contrast in the energy profiles of the one-state (black curve) and two-state (red curve) systems suggests distinct self-assembly behaviors. The one-state system's path to the target is characterized by its smoother energy reduction, quick assembly process, and greater stability, reflecting a less complex energetic landscape. Conversely, the two-state system's pathway is marked by higher energy fluctuations, indicative of a richly detailed process with multiple intermediate states. These findings illuminate the nuanced differences in self-assembly dynamics, providing insights into the thermodynamic efficiencies of these systems under the influence of external driving forces.

S5 External drive effects in self-assembly of large systems

To demonstrate the effectiveness of our proposed design principle in overcoming equilibrium constraints in large systems, we present both equilibrium and nonequilibrium MC simulations of 1000 patchy particles. The patches in this simulation are parameterized similarly to those in the smaller system of 8 patchy particles. While our previous analyses were limited to small-scale systems, it is crucial to extend this investigation to larger systems to assess the impact of the external drive. From the large-scale simulation, we observed a diverse ensemble of rings in various sizes, along with chains of different lengths. This prompted us to compute three key quantities, including the average number of bonds formed by each patchy particle ($\overline{N_B}$), the total number of rings formed per MC step (N_R), and the distribution of ring sizes. These quantities were computed over the last 1×10^6 MC steps, both in equilibrium across a range of ϵ_{patch} values and in nonequilibrium over a comparable range of ϵ_{drive} for selected ϵ_{patch} values. The ranges used here are consistent with those employed in the small-scale system.

In Fig. S8, we show the variation of $\overline{N_B}$ and N_R, respectively, as a function of ϵ_{patch} obtained from equilibrium simulations. Each plot includes two sets of data: one for particles

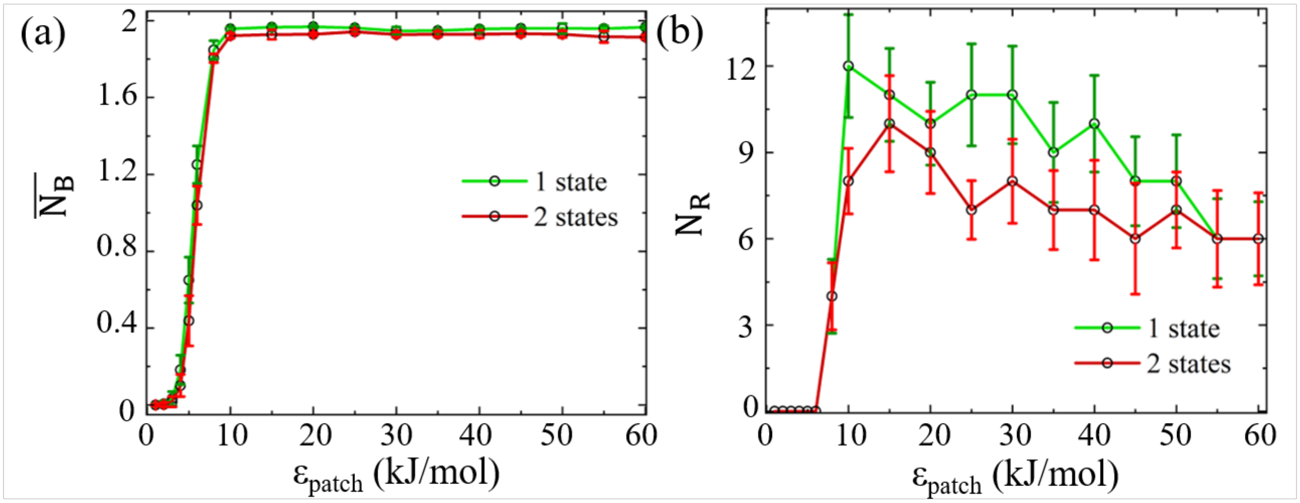

Figure S8: Equilibrium MC simulation results of the large system of 1000 particles: (a) Average number of bonds per particle, $\overline{N_B}$ and (b) Average number of rings, N_R per MC step as a function of patchy interaction energy is shown over the last 1×10^6 MC steps from equilibrium for both one and two-state systems. The error bar represents the standard deviation.

with a single state (green line) and another for particles with two states (red line). As ϵ_{patch} increases from 0 to 8 kJ/mol, there is a rapid rise in $\overline{N_B}$ from 0 to approximately $1.7 - 1.8$ for both types of systems. Beyond this point, $\overline{N_B}$ plateaus at around 2 bonds per particle at higher interaction energies, indicating that nearly all possible bonds are formed (Fig. S8(a)). As it is not evident from $\overline{N_B}$ alone how the system configuration changes in the due course of simulations, N_R is computed and plotted in see Fig. S8(b). It is seen that no rings are formed at lower ϵ_{patch} values ($0-6$ kJ/mol). As ϵ_{patch} increases, N_R rises to about $9-12$ rings in the one-state system, and $6 - 9$ rings in the two-state system, within the ϵ_{patch} range of $10 - 35$ kJ/mol. However, as ϵ_{patch} increases further up to 60 kJ/mol, the value of N_R decreases. The behavior of N_R with an increase in ϵ_{patch} closely resembles the variation observed in T_{fas} for the smaller system of 8 patchy particles (see Fig. 2(a) and 2(b) of the main manuscript). In Region I (low ϵ_{patch}), T_{fas} is high with no ring formation. Region II (medium ϵ_{patch}) depicts lower T_{fas} and maximum ring formation, while Region III (high ϵ_{patch}), a rise in T_{fas} with fewer rings formation.

Fig. S9 shows the distribution of ring sizes as a function of the patch interaction energy

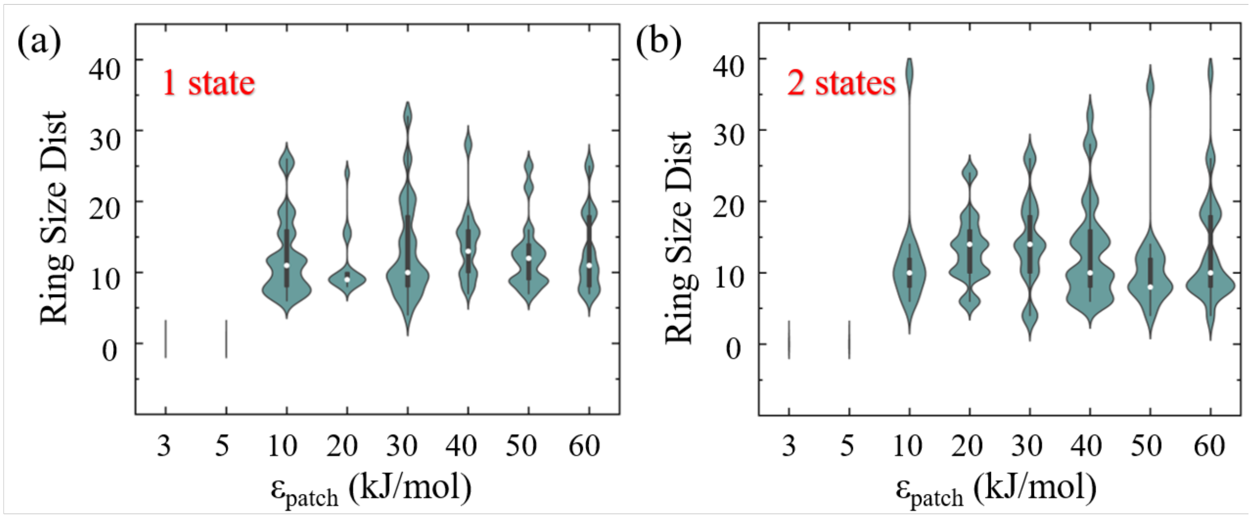

Figure S9: Equilibrium MC simulation results of a large system with 1000 particles: Ring size distribution over the last 1×10^6 MC steps as a function of patch interaction energy (ϵ_{patch}) is shown for both (a) one-state and (b) two-state systems. The circular dots represent the median, while the black horizontal lines indicate the 1.5 interquartile range. Data points beyond this range are considered outliers.

(ϵ_{patch}) for both single and two-state systems in equilibrium MC simulations. At lower ϵ_{patch} values (3 and 5 kJ/mol from region I), no rings are formed. At larger ϵ_{patch} values, rings of various sizes ranging from 8 particles to approximately 20 particles are formed, with a broader distribution around the smaller ring sizes. In the one-state system (see Fig. S9(a)), the ring sizes are primarily concentrated in the range of 9 to 12 particles across all ϵ_{patch} values, while in the two-state system (see Fig. S9(b)), the ring sizes formed are in the range of 9 to 15 particles.

Fig. S10 presents nonequilibrium MC simulation results, showing the variation of $\overline{N_B}$ and N_R as a function of increasing drive energy (ϵ_{drive}) for single-state and two-state systems at patch interaction energies ϵ_{patch} = 3 and 5 kJ/mol. In the single-state system (Fig. S10 (a)), $\overline{N_B}$ increases with ϵ_{drive} for both ϵ_{patch} values. Bonding gradually saturates at ϵ_{patch} = 3 kJ/mol, while at ϵ_{patch} = 5 kJ/mol, bonding increases quickly, indicating faster bond maximization. The two-state system (Fig. S10 (b)) shows a similar trend, but bonding saturates more gradually for both the values of ϵ_{patch}. In the single-state system (Fig. S10 (c)), N_R increases with ϵ_{drive} for both ϵ_{patch} values, with a more gradual rise at ϵ_{patch} = 3

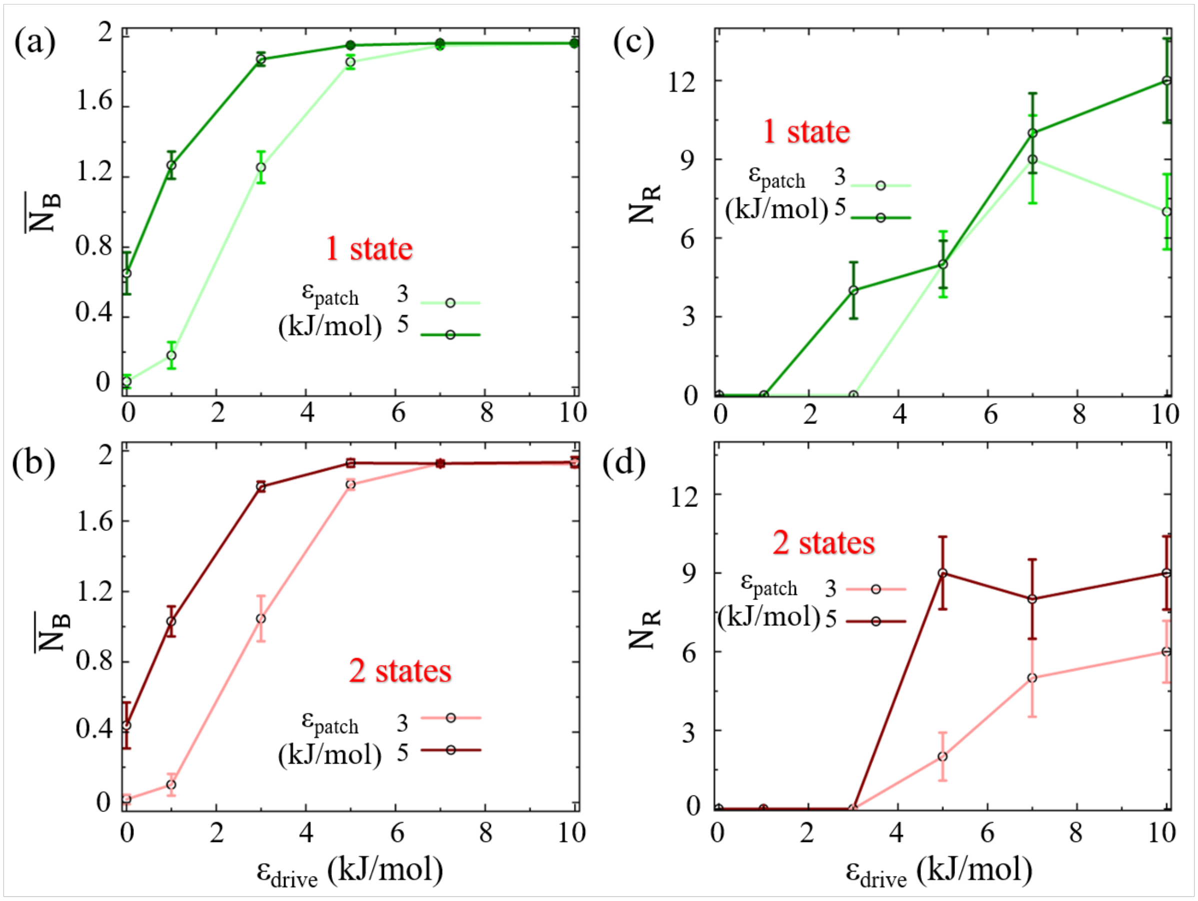

Figure S10: Nonequilibrium MC simulation results of a large system with 1000 particles for ϵ_{patch} value of 3 and 5 kJ/mol. $\overline{N_B}$ is plotted as a function of ϵ_{drive} for (a) one-state and (b) two-state system, N_R is plotted as a function of patchy interaction energy for (c) one-state and (d) two-state system. The error bar represents the standard deviation.

kJ/mol, reflecting a slower response to the drive energy. For $\epsilon_{patch} = 5$ kJ/mol, ring formation is faster, indicating stronger patch interactions enhance ring formation. In the two-state system (Fig. S10 (d)), N_R also rises with ϵ_{drive}. Compared to the one-state system, the value of N_R decreases for the two-state system, as expected and observed throughout our study.

Fig. S11 shows nonequilibrium MC simulation results of the ring size distribution as a function of increasing drive energy (ϵ_{drive}) for the one-state and two-state system at $\epsilon_{patch} = 3$ and 5 kJ/mol. At $\epsilon_{patch} = 3$ kJ/mol and lower drive energies, no rings are formed (Fig. S11 (a)), whereas for ϵ_{drive} values beyond 3 kJ/mol, ring formation begins. At $\epsilon_{patch} = 5$ kJ/mol, there is a similar trend, but ring formation occurs at a lower drive energy compared to the 3

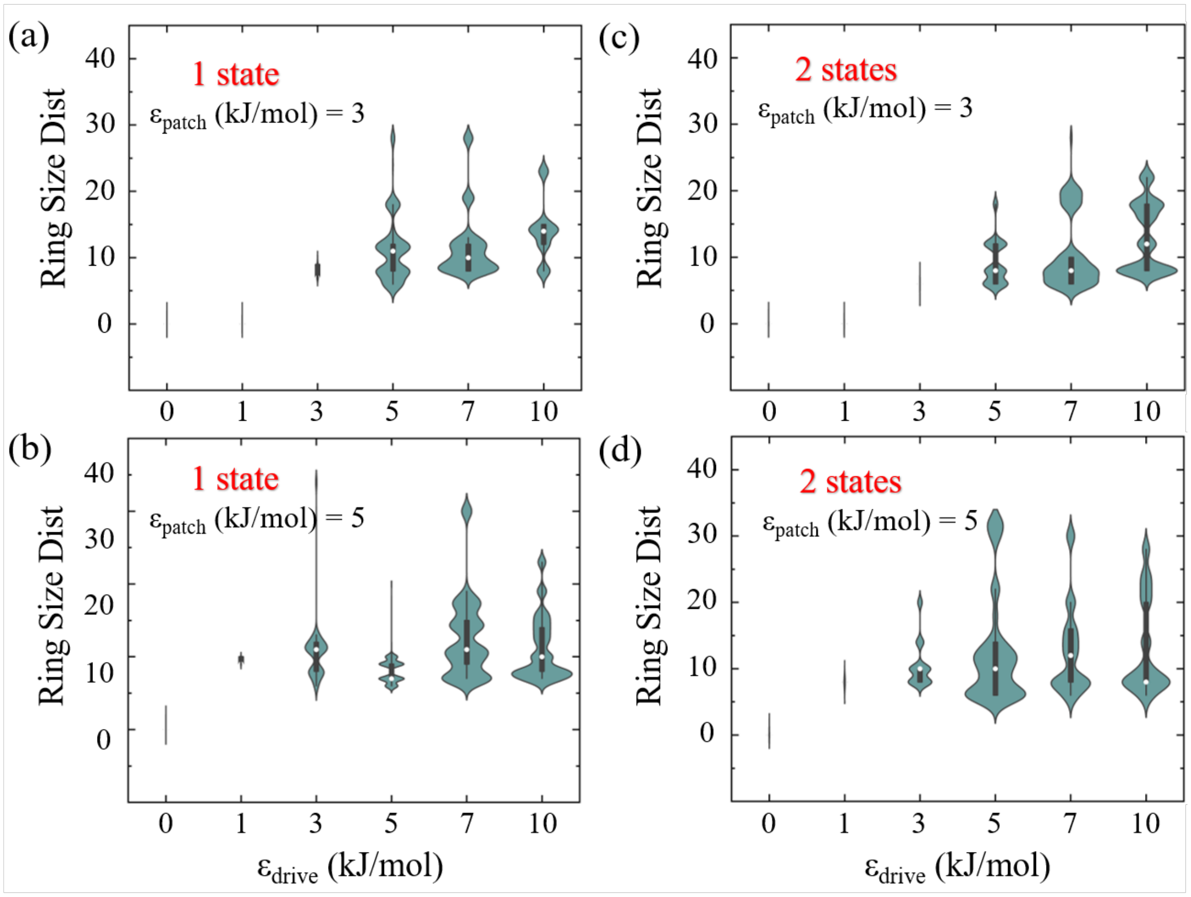

Figure S11: Nonequilibrium MC simulation results of a large system with 1000 particles for ϵ_{patch} value of 3 and 5 kJ/mol. Ring size distribution is shown over a range of ϵ_{drive} for (a) ϵ_{patch} = 3 kJ/mol and one state. (b) ϵ_{patch} = 5 kJ/mol and one states. (c) ϵ_{patch} = 3 kJ/mol and two state. (d) ϵ_{patch} = 5 kJ/mol and two states. The circular dots represent the median, while the black horizontal lines indicate the 1.5 interquartile range. Data points beyond this range are considered outliers.

kJ/mol case, specifically around rings of $8-9$ particles. (Fig. S11 (b)). A similar observation is evident for two-state systems as well (see Fig. S11 (c) and (d)). These analyses demonstrate that introducing an external drive significantly enhances the assembly of the system in both single-state and two-state configurations.

Fig. S12 illustrates the variation of $\overline{N_B}$ as a function of MC steps for different values of the external drive and patch interaction energy. At equilibrium ($\epsilon_{drive} = 0$ kJ/mol), the system forms only a few bonds. When the drive is slightly increased, ranging from approximately 5 kJ/mol for $\epsilon_{patch} = 3$ kJ/mol and 3 kJ/mol for $\epsilon_{patch} = 5$ kJ/mol, the $\overline{N_B}$ value rises to

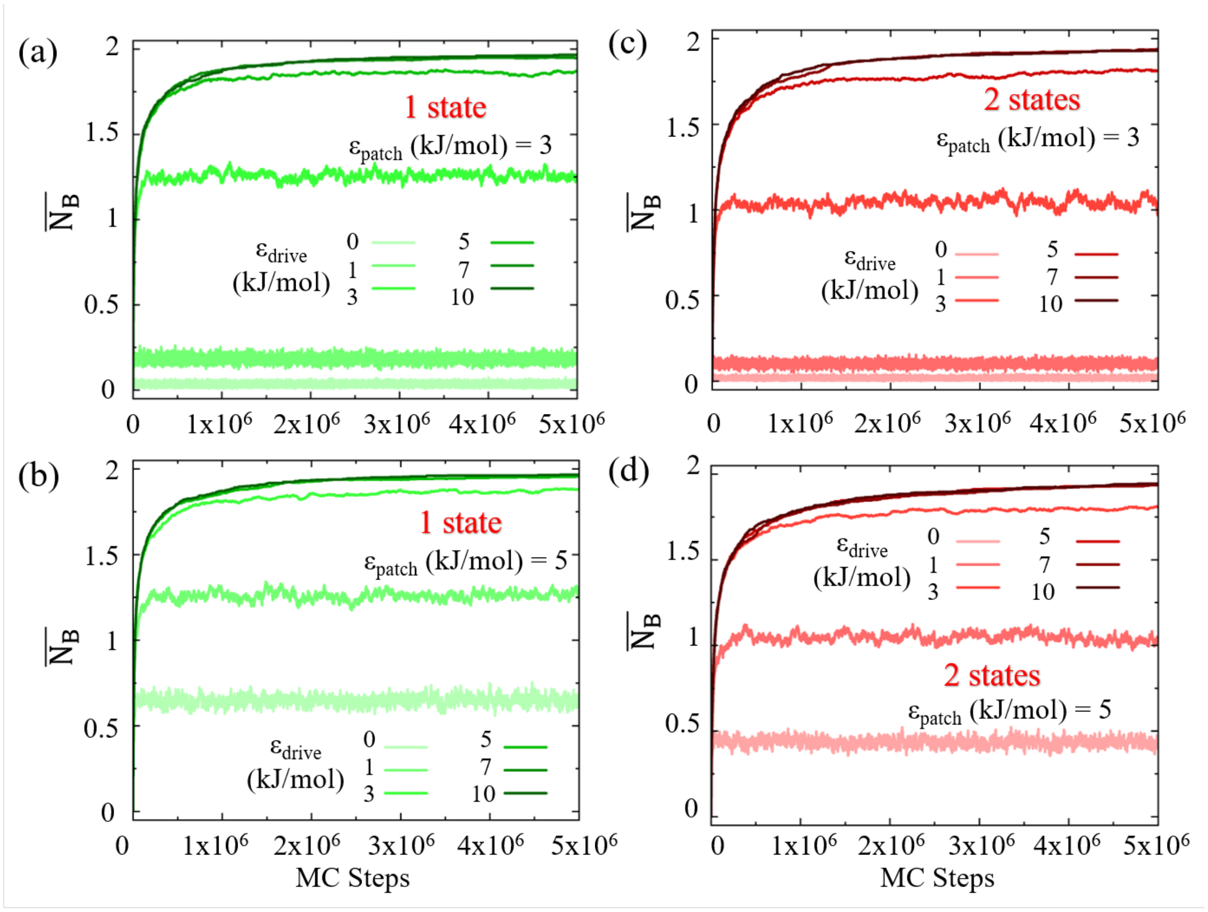

Figure S12: Nonequilibrium MC simulation results of the large system of 1000 particles. Variation in $\overline{N_B}$, as a function of the MC steps at different ϵ_{drive} values of 0 (equilibrium), 1, 3, 5, 7, and 10 kJ/mol for different ϵ_{patch} values and different systems. (a) $\epsilon_{patch} = 3$ kJ/mol and one state. (b) $\epsilon_{patch} = 5$ kJ/mol and one state. (c) $\epsilon_{patch} = 3$ kJ/mol and two states. (d) $\epsilon_{patch} = 5$ kJ/mol and two states.

the value of ~ 1.2, indicating that each particle forms at least one bond. This suggests the presence of chains (or very few rings) that have not yet stabilized. With a further increase in the drive energy, the $\overline{N_B}$ value rapidly approaches $1.8 - 1.9$, indicating that most particles have 2 bonds, leading to the stabilization of the desired structures.

Fig. S13 presents snapshots illustrating the final configurations from equilibrium and nonequilibrium simulations of a large-scale system with particles with two states. In Fig. S13 (a), where the ϵ_{patch} is set to 5 kJ/mol, the system remains in a disordered state, with particles randomly distributed throughout the simulation box and no apparent formation of organized structures like rings (see Movie S5). This indicates that, in the absence of an

external drive, the patch interactions alone are insufficient to induce significant self-assembly. In contrast, Fig. S13 (b) shows a system under the same patch interaction energy but with an applied drive energy of 10 kJ/mol (see Movie S6). Introducing this drive energy results in the formation of well-defined ring structures, highlighted by red circles. This emphasizes the critical role of the external drive in promoting self-assembly and structuring within the system, transforming it from a disordered state to an organized configuration.

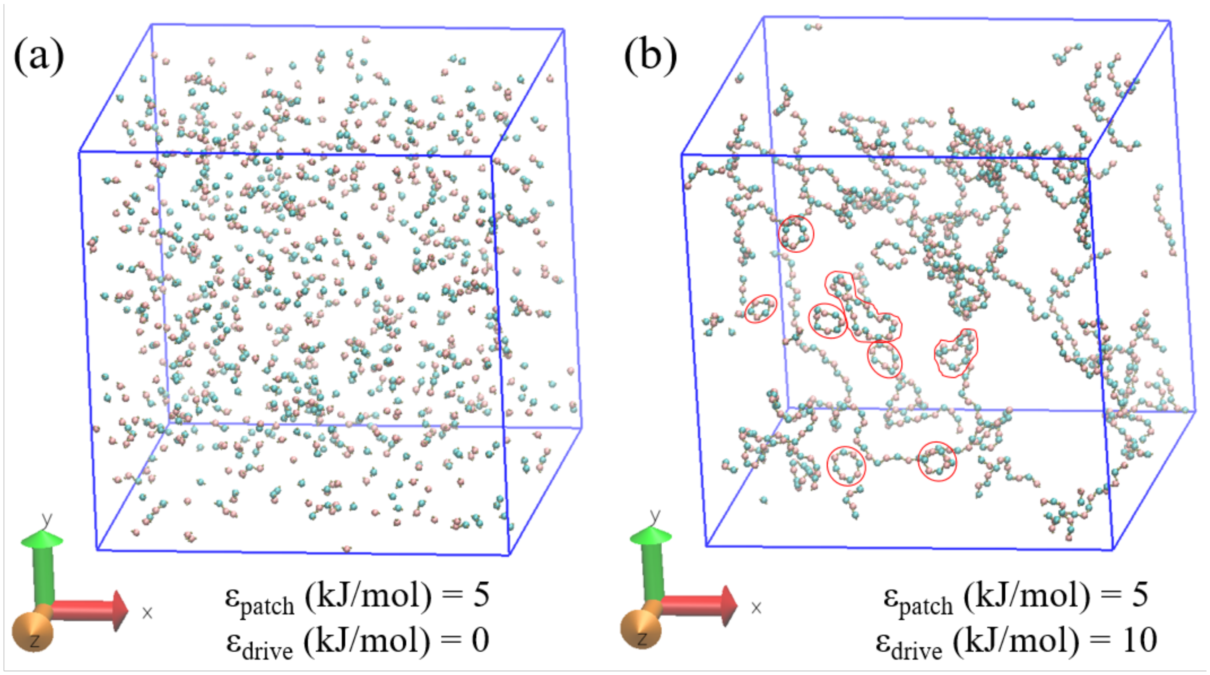

Figure S13: Snapshots of the final configuration from the simulation of a large-scale two-state system: (a) $\epsilon_{patch} = 5$ kJ/mol, $\epsilon_{drive} = 0$ kJ/mol. (b) $\epsilon_{patch} = 5$ kJ/mol, $\epsilon_{drive} = 10$ kJ/mol. Ring structures formed in (b) are highlighted with red circles.

S6 Effect of the square wave of periodicity on MD simulation results

In this section, we explore the impact of the periodicity of the external, time-dependent square wave potentials on the self-assembly kinetics (T_{fas}) of 8 patchy particles within the crowded environment using MD simulations. Fig. S14 illustrates the interaction energy, with

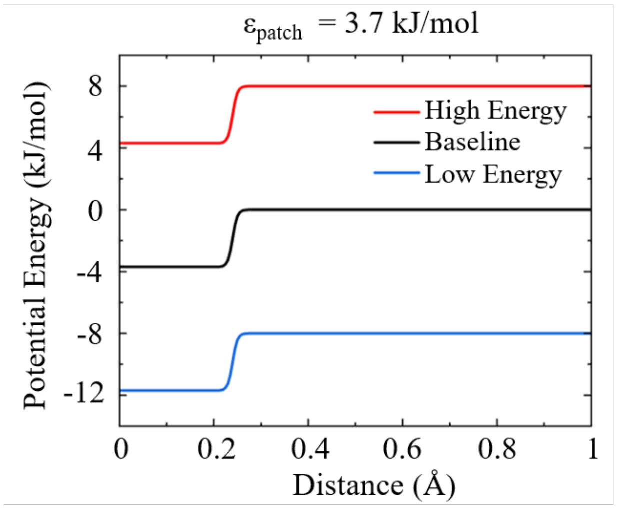

Figure S14: The patchy interaction potential of 3.7 kJ/mol for the high energy phase of the square wave potential (red), the low energy phase of the square wave potential (blue), and the baseline value (black). The amplitude of the square wave potential is 8 kJ/mol.

a baseline of 3.7 kJ/mol, and the high and low energy phases of the square wave potential, each with an amplitude of 8 kJ/mol. To complement the results presented in the main text for alternation between the high and low potential with a frequency of 20 ps, we extend our simulation to a periodicity of 40 ps to study the effect of the potential switching frequency on the results.

The median first self-assembly times, T_{fas}, show an overall decrease with increasing amplitude of the square wave potential (Fig. S15). Notably, the distribution of results of individual realizations underscores the stochastic nature of the self-assembly process.

Comparing the effects of a 40 ps periodicity with those documented for a 20 ps periodicity (Fig. 5(b) in the main text), the reduction in the time to first assembly (T_{fas}) is less substantial at 40 ps. This difference can be attributed to the kinetic impact of the more frequent application of external forces for shorter periodicity, facilitating bond formation.

This relationship between the periodicity of the external driving force and the system kinetic response is crucial for the strategic application of external controls during the self-assembly process. Consequently, identifying an optimal periodicity is essential for precisely modulating assembly rates in patchy particle systems under nonequilibrium conditions, high-

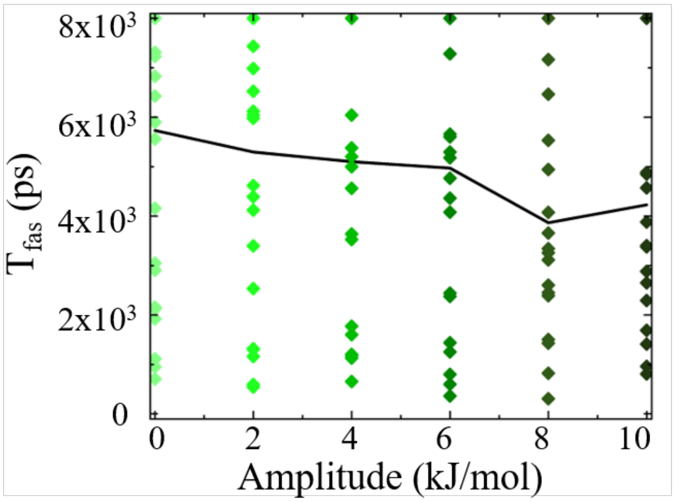

Figure S15: Median T_{fas} as a function of the amplitude of square-wave potential (periodicity: 40 ps) during nonequilibrium MD simulations within a crowded environment at $\epsilon_{patch} = 3.7$ kJ/mol. Individual realizations are indicated by diamonds, with the median value traced by a black curve.

lighting its importance in the design and execution of self-assembly experiments.

To further demonstrate the robustness and consistency of using a periodic square wave potential external drive, we extended our study to include two-state systems with crowding agents. The simulations were carried out using the same parameters as the crowd simulations carried out with one state, except the patchy particles were now modeled to form rings with neighbors of opposite states, similar to Fig. 1 of the main text. In the MC simulations, we observed a similar reduction in T_{fas} from 7×10^6 to $\sim 2 \times 10^5$ with increasing driving forces (Fig. S16(a)), as seen in the one-state system. This indicates faster assembly when the external bias increases from 0 kJ/mol to higher values. Similarly, in the MD simulations, we used the periodic wave previously applied to a crowded system with one state (see Fig. S16(b)). In equilibrium, the system could not assemble within the given simulation length. With an increase in the amplitude of the square wave to 4 kJ/mol, T_{fas} started to decrease from the simulation length, indicating that most of the simulations were now assembling compared to lower or zero external drives. With a further increase in the external potential to an amplitude of 10 kJ/mol, we observed that T_{fas} decreased to 5893 ps from

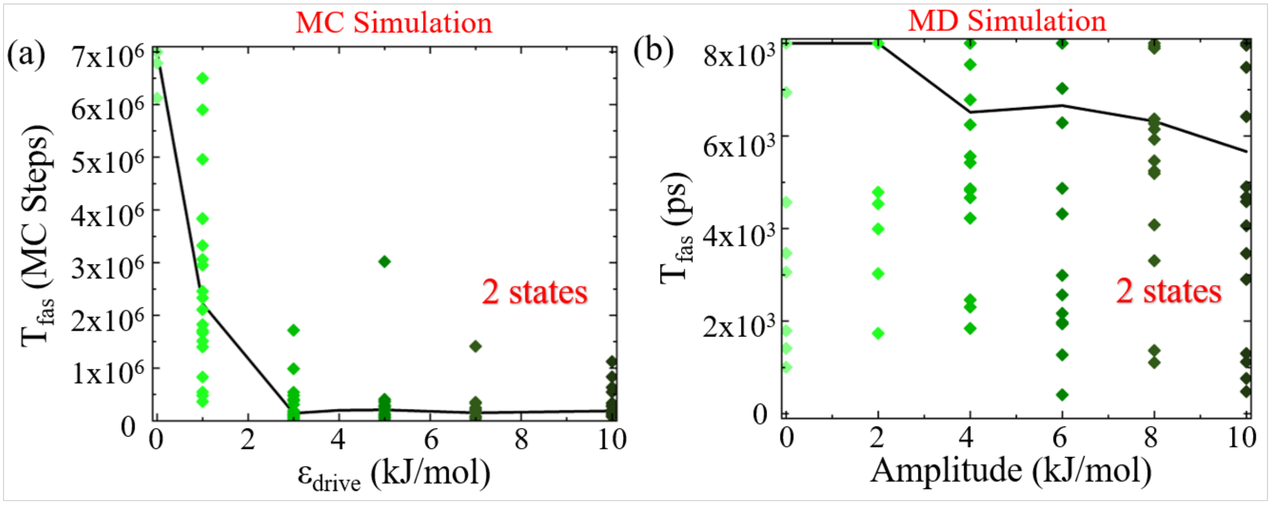

Figure S16: Crowd simulation with patchy particles of two states. (a) In MC simulations, the variation of T_{fas} is displayed as a function of the external drive value. (b) In MD simulations, this variation is displayed as a function of the amplitude of the external time-dependent square potential with a periodicity of 20 ps. A single realization is represented by each dot, and the solid black line shows the median value.

8000 ps. Both the MC and MD simulation observations are consistent with our previous findings in the one-state 8-particle system with crowding agents shown in Fig. 5 of the main text (see Movie S9 for the nonequilibrium realizations).

In order to gain a better understanding of bond formation dynamics, we ran MD simulations of a system with 2 patchy particles. These simulations were initiated randomly and continued until the first bond formation event occurred, under both equilibrium and nonequilibrium conditions. We monitored the total potential energy and the force between the particles. The simulation cell had dimensions of $3 \times 6 \times 6$ Å^3. All other simulation parameters were consistent with those used in the 8 patchy particle systems of one state with 48 crowds. It is worth noting that the results were not strictly dependent on the simulation cell dimensions or patch parameters ($\delta/2$ and θ_{max}).

Under equilibrium conditions with patchy interaction energies, ϵ_{patch}, of 3.7 kJ/mol (Fig. S17(a)), the potential energy initially fluctuates around 0, and the force between the particles also remains close to 0. At 312 ps, a bond forms, leading to a noticeable decrease in the potential energy and a spike in the force. In contrast, non-equilibrium conditions

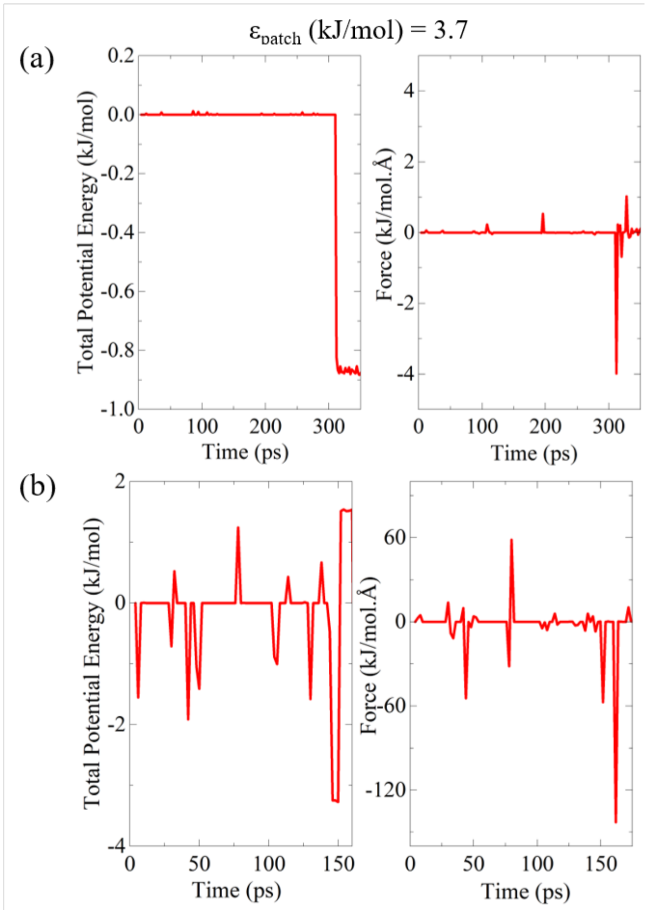

Figure S17: MD simulation of a system of 2 patchy particles up to the first bond formation event. Total potential energy and force as a function of time for (a) equilibrium conditions with ϵ_{patch} value of 3.7 kJ/mol and (b) nonequilibrium conditions with square wave potential amplitude of 8 kJ/mol for ϵ_{patch} value of (b) 3.7 kJ/mol, shown for individual realizations.

show a different total potential energy and force profile (see Fig. S17(b)). In this case, the potential energy fluctuates significantly due to varying high and low energy phases of the square wave potential. As a result, larger forces act between the two particles compared to the equilibrium values. This causes the particles to experience frequent and substantial changes in energy, compelling them to reorient and adjust their positions as they seek stable configurations. The difference in force magnitudes between equilibrium and nonequilibrium emphasizes the mechanism of increased bond-formation events. In the nonequilibrium case, these larger forces accelerate the bonding process.

S7 Supplementary movies

Movie S1: This movie presents an equilibrium MC simulation of 8 patchy particles, with 1 internal state, contained within a cubic box of 20 Å side length with reflective boundaries. The simulation employs an interaction energy of 4 kJ/mol for interactions between patches and does not incorporate any external driving forces. Conducted at a temperature of 65 K, the simulation spans 7×10^6 MC steps, with the trajectory recorded every 1000th step.

Movie S2: This movie details an equilibrium MC simulation of 8 patchy particles, with 2 internal states, confined within a cubic box of 20 Å in length and featuring reflective boundaries. The simulation utilizes an interaction energy of 4 kJ/mol among the patches, with no external driving forces applied. It is conducted at a temperature of 65 K over 7×10^6 MC steps, with the trajectory being captured every 1000th MC step.

Movie S3: This movie showcases a nonequilibrium MC simulation of 8 patchy particles, with 1 internal state, contained within a cubic box of 20 Å side length and reflective boundaries. This simulation was executed with an interaction energy of 4 kJ/mol between the patches, under the influence of an external driving force of 7 kJ/mol. Performed at a temperature of 65 K, the simulation extends over 7×10^6 MC steps, with the trajectory being recorded every 1000th step.

Movie S4: This movie presents a nonequilibrium MC simulation of 8 patchy particles, with 2 internal states, enclosed in a cubic box of 20 Å side length with reflective boundaries. The simulation utilizes an interaction energy of 4 kJ/mol between patches, alongside an external driving force of 7 kJ/mol. Conducted at 65 K, the simulation proceeds for 7×10^6 MC steps, with trajectory data recorded every 1000th step.

Movie S5: This movie presents an equilibrium MC simulation of 1000 patchy particles, with 2 internal states, contained within a cubic box of 100 Å side length with periodic boundaries. The simulation employs an interaction energy of 5 kJ/mol for interactions between patches and does not incorporate any external driving forces. Conducted at a temperature of 65 K, the simulation spans 5×10^6 MC steps, with the trajectory being

recorded every 500th step.

Movie S6: This movie showcases a nonequilibrium MC simulation of 1000 patchy particles, with 2 internal states, contained within a cubic box of 100 Å side length and periodic boundaries. This simulation was executed with an interaction energy of 5 kJ/mol between the patches, under the influence of an external driving force of 10 kJ/mol. Performed at a temperature of 65 K, the simulation extends over 5×10^6 MC steps, with the trajectory being recorded every 500th step.

Movie S7: This movie demonstrates MD simulation of 8 patchy particles with 1 internal state, accompanied by 48 crowding agents, within a cubic box of 10 Å in length with periodic boundaries. In equilibrium conditions, the simulation was executed with an interaction energy of 3.7 kJ/mol between the patches. It is performed at 40 K for 8 ns, with the trajectory being recorded every 2 ps. While the van der Waals radii of the patchy particle beads and the crowding agents are identical, for improved visualization of target formation, the crowding agents' size in the movie is reduced by 33% relative to the bead.

Movie S8: This movie showcases nonequilibrium MD simulation of 8 patchy particles with 1 internal states and 48 crowding agents within a cubic box measuring 10 Å in length with periodic boundaries. This simulation in nonequilibrium was conducted with an interaction energy of 3.7 kJ/mol between the patches, employing a time-dependent square wave of periodicity 20 ps at an amplitude of 10 kJ/mol. The simulation is run at 40 K for 8 ns simulation length. During the simulation, the trajectory is stored every 2 ps. The same adjustment to the size of crowding agents as implemented in Movie S7 has been applied here as well.

Movie S9: This movie showcases a nonequilibrium MD simulation of 8 patchy particles with 2 internal states and 48 crowding agents within a cubic box measuring 10 Å in length with periodic boundaries. This simulation in nonequilibrium was conducted with an interaction energy of 3.7 kJ/mol between the patches, employing a time-dependent square wave of periodicity 20 ps at an amplitude of 6 kJ/mol. The simulation is run at 40 K for 8

ns simulation length. During the simulation, the trajectory is stored every 2 ps. The same adjustment to the size of crowding agents as implemented in Movie S7 has been applied here as well.

References

(1) Kern, N.; Frenkel, D. Fluid–fluid coexistence in colloidal systems with short-ranged strongly directional attraction. *J. Chem. Phys.* **2003**, *118*, 9882–9889.

(2) Zhang; Keys, A. S.; Chen, T.; Glotzer, S. C. Self-Assembly of Patchy Particles into Diamond Structures through Molecular Mimicry. *Langmuir* **2005**, *21*, 11547–11551.

(3) Reinhart, W. F.; Panagiotopoulos, A. Z. Equilibrium crystal phases of triblock Janus colloids. *J. Chem. Phys.* **2016**, *145*, 094505.

(4) Neophytou, A.; Chakrabarti, D.; Sciortino, F. Facile self-assembly of colloidal diamond from tetrahedral patchy particles via ring selection. *Proc. Natl. Acad. Sci. U. S. A.* **2021**, *118*, e2109776118.

(5) Beneduce, C.; E. P. Pinto, D.; Šulc, P.; Sciortino, F.; Russo, J. Two-step nucleation in a binary mixture of patchy particles. *J. Chem. Phys.* **2023**, *158*, 154502.

(6) Smallenburg, F.; Sciortino, F. Liquids more stable than crystals in particles with limited valence and flexible bonds. *Nat. Phys.* **2013**, *9*, 554–558.

(7) Rovigatti, L.; Russo, J.; Romano, F. How to simulate patchy particles. *Eur. Phys. J. E* **2018**, *41*, 59.

(8) Metropolis, N.; Rosenbluth, A. W.; Rosenbluth, M. N.; Teller, A. H.; Teller, E. Equation of State Calculations by Fast Computing Machines. *J. Chem. Phys.* **1953**, *21*, 1087–1092.

(9) Bisker, G.; England, J. L. Nonequilibrium associative retrieval of multiple stored self-assembly targets. *Proc. Natl. Acad. Sci. U. S. A.* **2018**, *115*, E10531–E10538.

(10) Verlet, L. Computer "Experiments" on Classical Fluids. I. Thermodynamical Properties of Lennard-Jones Molecules. *Phys. Rev.* **1967**, *159*, 98–103.

(11) Thompson, A. P.; Aktulga, H. M.; Berger, R.; Bolintineanu, D. S.; Brown, W. M.; Crozier, P. S.; in 't Veld, P. J.; Kohlmeyer, A.; Moore, S. G.; Nguyen, T. D.; Shan, R.; Stevens, M. J.; Tranchida, J.; Trott, C.; Plimpton, S. J. LAMMPS - a flexible simulation tool for particle-based materials modeling at the atomic, meso, and continuum scales. *Comput. Phys. Commun.* **2022**, *271*, 108171.

(12) Nosé, S. A unified formulation of the constant temperature molecular dynamics methods. *J. Chem. Phys.* **1984**, *81*, 511–519.

(13) Hoover, W. G. Canonical dynamics: Equilibrium phase-space distributions. *Phys. Rev. A* **1985**, *31*, 1695–1697.

(14) Jover, J.; Haslam, A. J.; Galindo, A.; Jackson, G.; Müller, E. A. Pseudo hard-sphere potential for use in continuous molecular-dynamics simulation of spherical and chain molecules. *J. Chem. Phys.* **2012**, *137*, 144505.

(15) Espinosa, J. R.; Garaizar, A.; Vega, C.; Frenkel, D.; Collepardo-Guevara, R. Breakdown of the law of rectilinear diameter and related surprises in the liquid-vapor coexistence in systems of patchy particles. *J. Chem. Phys.* **2019**, *150*, 224510.

(16) Espinosa, J. R.; Vega, C.; Sanz, E. The mold integration method for the calculation of the crystal-fluid interfacial free energy from simulations. *J. Chem. Phys.* **2014**, *141*, 134709.

(17) Lecoultre, S.; Rydlo, A.; Félix, C.; Buttet, J.; Gilb, S.; Harbich, W. UV–visible absorption of small gold clusters in neon: Aun (n = 1–5 and 7–9). *J. Chem. Phys.* **2011**, *134*, 074302.

(18) Terlau, H.; Stühmer, W. Structure and Function of Voltage-Gated Ion Channels. *Naturwissenschaften* **1998**, *85*, 437–444.

(19) Fang, X.; Liu, Q.; Bohrer, C.; Hensel, Z.; Han, W.; Wang, J.; Xiao, J. Cell fate potentials and switching kinetics uncovered in a classic bistable genetic switch. *Nat. Commun.* **2018**, *9*, 2787.

(20) Shields IV, C. W.; Zhu, S.; Yang, Y.; Bharti, B.; Liu, J.; Yellen, B. B.; Velev, O. D.; López, G. P. Field-directed assembly of patchy anisotropic microparticles with defined shape. *Soft Matter* **2013**, *9*, 9219–9229.

(21) Song, P.; Wang, Y.; Wang, Y.; Hollingsworth, A. D.; Weck, M.; Pine, D. J.; Ward, M. D. Patchy Particle Packing under Electric Fields. *J. Am. Chem. Soc.* **2015**, *137*, 3069–3075.

(22) Nag, S.; Bisker, G. Dissipative self-assembly of patchy particles under nonequilibrium drive: a computational study. *Submitted*

(23) Carlier, M.-F.; Gutfreund, H.; Bayley, P. M. Nucleotide Hydrolysis Regulates the Dynamics of Actin Filaments and Microtubules. *Philos. Trans. R. Soc., B* **1992**, *336*, 93–97.

(24) Piedra, F.-A.; Kim, T.; Garza, E. S.; Geyer, E. A.; Burns, A.; Ye, X.; Rice, L. M. GDP-to-GTP exchange on the microtubule end can contribute to the frequency of catastrophe. *Mol. Biol. Cell* **2016**, *27*, 3515–3525.

(25) Stenhammar, J.; Wittkowski, R.; Marenduzzo, D.; Cates, M. E. Light-induced self-assembly of active rectification devices. *Sci. Adv.* **2016**, *2*, e1501850.

(26) Das, K.; Gabrielli, L.; Prins, L. J. Chemically Fueled Self-Assembly in Biology and Chemistry. *Angew. Chem., Int. Ed.* **2021**, *60*, 20120–20143.

(27) Lin, T.; Wu, Q.; Liu, J.; Shi, Z.; Liu, P. N.; Lin, N. Thermodynamic versus kinetic control in self-assembly of zero-, one-, quasi-two-, and two-dimensional metal-organic coordination structures. *J. Chem. Phys.* **2015**, *142*, 101909.
